# Author correction: Association between exposure to particulate matter during pregnancy and birthweight: A systematic review and a meta-analysis of birth cohort studies

**DOI:** 10.7555/JBR.38.20240383

**Published:** 2025-07-16

**Authors:** Yinwen Ji, Fei Song, Bo Xu, Yining Zhu, Chuncheng Lu, Yankai Xia

**Affiliations:** 1 State Key Laboratory of Reproductive Medicine, Institute of Toxicology, Nanjing Medical University, Nanjing, Jiangsu 211166, China; 2 Key Laboratory of Modern Toxicology of Ministry of Education, School of Public Health, Nanjing Medical University, Nanjing, Jiangsu 211166, China; 3 Department of Research and Education, The Children's Hospital, Zhejiang University School of Medicine, Hangzhou, Zhejiang 310003, China; 4 Department of Epidemiology and Biostatistics, School of Public Health, Tianjin Medical University, Tianjin 300070, China; 5 Department of Thoracic Surgery, The First School of Clinical Medicine, Nanjing Medical University, Nanjing, Jiangsu 210029, China

**Keywords:** PM_2.5_, PM_10_, birth weight, cohort study, meta-analysis

## Abstract

The effect of prenatal exposure to ambient particulate matter (PM) on birth weight varies considerably across studies, and the findings remain inconclusive. In this study, we conducted a meta-analysis to assess the associations between exposure to PM_2.5_ and PM_10_ and birth weight. A total of 74 studies were identified through searches in Web of Science, PubMed, Embase, and Ovid Medline, as well as manual searches, up to October 2024. We found that for each 10 μg/m³ increase in PM_2.5_, the risk of low birth weight (LBW) increased significantly during the entire pregnancy (odds ratio [OR] = 2.41, 95% confidence interval [CI]: 1.99–2.91) and in all trimesters. Similarly, for every 10 μg/m³ increase in PM_10_ concentration, the risk of LBW increased significantly during the entire pregnancy (OR = 1.46, 95% CI: 1.16–1.84). Subgroup analysis by maternal age for PM_2.5_ showed that mothers aged 30 and above had a significantly higher risk of LBW (OR = 3.69, 95% CI: 2.81–4.84), compared with those under 30. In conclusion, maternal exposure to PM_2.5_ and PM_10_ is associated with an increased risk of LBW across all trimesters. Additionally, mothers aged 30 and above are at a higher risk of LBW, compared with younger mothers. Further research is needed to clarify the biological mechanisms by which PM pollution may contribute to LBW.

## Introduction

Ambient particulate matter (PM) is considered one of the most harmful air pollutants, and it is widely recognized as a major risk factor for global health, significantly contributing to the worldwide disease burden and posing a critical public health challenge^[1]^. In 2019, exposure to PM_2.5_ was associated with over 4.1 million deaths globally, representing a 102.3% increase compared with the past three decades^[2]^. Moreover, for every 10 μg/m^3^ increase in PM_10_ levels, there was a corresponding 0.44% increase in overall mortality rates^[3]^. In China, PM has been gaining increasing attention as a significant public health concern. Most Chinese individuals are exposed to concentrations well above the thresholds suggested by the World Health Organization (WHO)^[4]^. Chronic exposure to PM_2.5_ is associated with various adverse health outcomes, including respiratory diseases, cardiovascular diseases, and adverse pregnancy outcomes^[5–7]^.

Pregnancy constitutes a critical period during which women are particularly vulnerable to environmental factors^[8–9]^. Birth weight, a key indicator of fetal growth and development, is a crucial outcome of pregnancy^[10]^. Low birth weight (LBW), defined as a birth weight of less than 2500 g, is considered an adverse birth outcome associated with both short- and long-term health impacts, such as growth retardation, metabolic syndrome, and cognitive impairments^[11–14]^. Therefore, it is essential to identify factors that affect an infant's birth weight. Studies have investigated the correlation between gestational exposure to PM_2.5_ and newborn birth weight. However, findings remain inconclusive. For example, Liang *et al*^[15]^ reported that exposure to PM_2.5_ throughout gestation increased the risk of LBW, and Lin *et al*^[16]^ similarly identified associations between exposure to both PM_2.5_ and PM_10_ and the risk of LBW. In contrast, Brauer *et al*^[17]^ found no significant association between PM_2.5_ or PM_10_ exposure and the risk of LBW. These discrepancies may be attributed to both the study design and the exposure assessment methods. Additionally, the timing of exposure to air pollutants during pregnancy also varies across studies, with some examining specific trimesters and others assessing exposure throughout the entire gestational period, contributing to the diverse findings reported.

Although several meta-analyses have assessed the impact of prenatal exposure to outdoor air pollution on adverse pregnancy outcomes, most focused on only one type of particulate matter. In this study, we aimed to quantitatively investigate the associations between exposure to PM_2.5_ and PM_10_ during pregnancy and birth weight, while also assessing the potential influence of factors such as maternal age and exposure assessment methods on these associations.

## Materials and methods

### Search strategy

A thorough search was performed using four major global databases of electronic literature (*i.e.*, Web of Science, PubMed, EMBASE, and Ovid MEDLINE) to gather all relevant manuscripts published before October 31, 2024, using the following search strategy: (pregnancy OR maternal OR gravidity) AND (particulate matter OR PM_2.5_ OR PM_10_ OR air pollution) AND (birth weight OR birthweight OR birth size OR birth outcome OR pregnancy outcome OR ponderal index OR small for gestational age OR large for gestational age OR macrosomia) AND (cohort OR observational OR longitudinal OR follow-up). In addition, we conducted a manual search of all references in relevant publications to identify additional studies and avoid omissions.

### Study selection

Studies meeting the inclusion criteria below were considered eligible: (1) Cohort study design; (2) Research focused on exposure to PM_2.5_ and PM_10_ during pregnancy; (3) Results reported as either continuous birth weight or binary LBW; (4) Results presented as regression coefficients (*β*), odds ratios (ORs), risk ratios (RRs), or hazard ratios (HRs) with 95% confidence intervals (CIs); and (5) Studies published in English. The exclusion criteria were as follows: (1) Conference proceedings, reviews, comments, books, and meta-analyses; (2) Animal studies; (3) Studies involving other exposure factors, or unrelated pregnancy outcomes; (4) Non-prenatal exposure; (5) No direct correlation between pollutants and birth weight; and (6) Low Newcastle-Ottawa Scale (NOS) score (> 5).

All retrieved documents were imported into EndNote X9 for screening. Initially, duplicates were automatically removed, followed by a manual review to ensure the exclusion of all remaining duplicates. Titles and abstracts of the literature were reviewed by two authors independently, based on the established criteria for inclusion and exclusion. For documents with unclear titles and abstracts, the full text and supplementary materials were reviewed to determine eligibility. The conflict between the two authors was resolved through discussion, and if necessary, a third investigator was consulted to achieve a consensus.

### Data extraction

Two authors independently extracted data from the studies included in the quantitative synthesis, with any discrepancies discussed and resolved through consensus. The extracted data comprised the following components: (1) Basic study information, including source (title and author), study period, study design, country of the cohort, and the sample size; (2) Air pollution concentrations (PM_2.5_ and PM_10_) and exposure periods (trimester-specific and/or entire pregnancy); (3) Effect size, including the regression coefficients, as well as OR/HR/RR and their 95% CIs, with effect estimates extracted from the fully adjusted model of each study; (4) Transformation methods, step units (*e.g.*, per 10 μg/m^3^) or interquartile range; (5) Exposure assessment methods, such as data from monitoring stations, land use regression (LUR) models, and inverse distance weighting (IDW) models; (6) Confounding factors considered in the analyses; (7) Outcomes of included studies; (8) NOS scores.

For studies that reported both single and multi-pollutant models, only the adjusted single-pollutant model was extracted. Additionally, in studies with subgroup analyses, we extracted the effect estimates for each subgroup. We also collected data on the impact of different exposure time windows.

### Quality assessment

The quality of the cohort studies included in our research was assessed using the NOS^[18]^. Only studies with scores > 5 were included in this study.

### Statistical analyses

A comprehensive meta-analysis of all included studies was conducted to estimate the pooled effects of maternal exposure to PM_2.5_ and PM_10_ during pregnancy and across the three trimesters on birthweight (*β*) and LBW (OR/HR/RR). Heterogeneity among estimates derived from primary studies was evaluated using the *I*^2^ statistic test. Low, moderate, and high heterogeneity were assigned to *I*^2^ values of 25%, 50%, and 75%, respectively. For those with high heterogeneity, the pooled ORs and corresponding 95% CIs were calculated using random-effects models.

Subgroup analyses were conducted according to the exposure windows, including the entire pregnancy, the first trimester (T1), the second trimester (T2), and the third trimester (T3). Additionally, to explore the possible sources of heterogeneity, we further performed subgroup meta-analyses, focusing on maternal age (< 30 *vs.* ≥ 30 years old) and exposure assessment methods, including aerosol optical depth (AOD), LUR, IDW, monitoring stations, and others. For subgroup analysis on maternal age, studies were divided into different subgroups based on the mean or median maternal age of the samples presented in the studies.

Given that 29 studies in the meta-analysis reported LBW outcomes, and that most effect sizes were expressed as ORs (21 studies), we approximated HRs and RRs as ORs for consistency^[19]^. All the *β* coefficients (95% CIs) for birth weight and ORs (95% CIs) for LBW were transformed into a standard unit of a 10 μg/m^3^ increase in PM_2.5_ or PM_10_ before carrying out the meta-analyses. This was achieved by using the following formula, substituting the initial step unit of fine particulate matter.

$ \mathit{\beta } _{ \mathrm{standardized}} \mathrm= \mathit{\beta } _{ \mathrm{initial}} \mathrm{/step}_{ \mathrm{initial}} {\times 10\;{\mug}/{\mathrm{m}}}^{ \mathrm{3}} $

$ \mathrm{OR}_{ \mathrm{standardized}} \mathrm{=OR}_{ \mathrm{initial}} \mathrm{/step}_{ \mathrm{initial}} \mathrm{\times 10\;{\mug}/m}^{ \mathrm{3}} $

Forest plots were used to visually present the distribution of effect sizes, along with their 95% CIs. Sensitivity analyses were conducted using the "leave-one-out" approach to assess the robustness of the results. Egger's, Begg's, and Thompson's tests were used to assess potential publication bias^[20]^. R software (version 4.4.1) was used for the entire statistical analysis. The "meta" package was installed and imported, with its "metagen" function used for analysis. *P*-values were assessed as two-tailed, with a threshold of less than 0.05 deemed statistically significant.

## Results

### Records included

***Fig. 1*** illustrates our literature search strategy. Initially, we identified 2521 records from PubMed, Embase, Web of Science, Ovid Medline, and other sources published before October 31, 2024. After removing duplicates, a total of 1388 studies remained. Of these, 1267 studies were excluded based on title or abstract screening because of their irrelevance to the research focus (*e.g.*, editorials, studies unrelated to our targeted health consequences, experiments on animals or cells). With a total of 121 articles potentially eligible, a full-text review was performed thoroughly. Ten of these were excluded based on the reasons outlined below: six were systematic reviews or meta-analyses, and four were conference abstracts. Among the remaining 111 studies, 37 were excluded during the final selection process: 29 investigated factors unrelated to birth weight, five examined non-prenatal exposures, and three failed to establish a direct association between pollutants and birth weight. Ultimately, our meta-analysis included 74 studies (***Fig. 1***). Among them, the meta-analysis for LBW included 59 articles, while the meta-analysis for birth weight included 22 articles, with seven articles included in both analyses.

**Figure 1:**
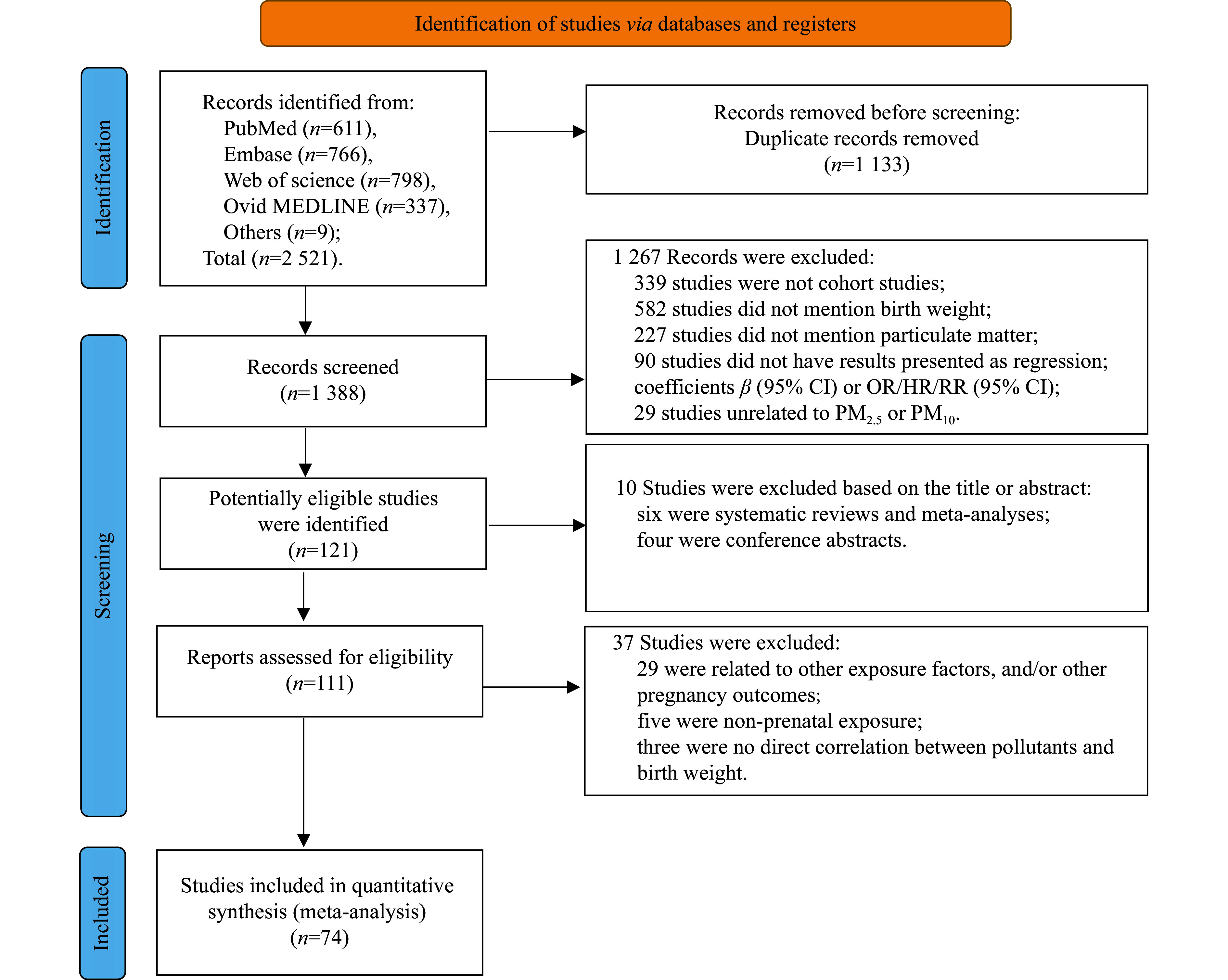
Flow chart of the study selection process. Abbreviations: CI, confidence interval; OR, odds ratio; HR, hazard ratio; RR, risk ratio; PM_2.5_, particulate matter with an aerodynamic diameter of 2.5 μm or less; PM_10_, particulate matter with an aerodynamic diameter of 10 μm or less.

### Characteristics of included studies

***Supplementary Table 1*** (available online) presents the attributes of the 74 cohort studies included in our meta-analysis. These studies encompassed a minimum of 340 births and a maximum of 3545177 births, totaling 28042477 births. Out of the 74 studies analyzed, 13 explored the association between PM_10_ and birth weight, while 39 examined the association between PM_2.5_ and birth weight. The remaining (*n* = 22) examined both. For exposure assessment methods, 23 studies used data from monitoring stations, 20 employed the LUR model, 10 used the IDW model (one study combined both methods), two used AOD-based models, and 18 studies used other models to measure exposure to PM_2.5_ or PM_10_. During the entire pregnancy, the average concentration of daily exposure to PM_2.5_ ranged from 1.1 μg/m^3^ to 228.5 μg/m^3^, with PM_10_ levels ranging from 3.3 μg/m^3^ to 116.49 μg/m^3^. In terms of exposure windows, 60 studies covered the entire pregnancy, seven assessed the exposure by trimester, and seven focused on one or two specific trimesters. Among the studies, 66 provided information on whether the mothers were aged 30 or older. More than 26 covariates were reported in these studies, with the most common being maternal age (*n* = 63), infant sex (*n* = 59), parity (*n* = 52), maternal smoking status (*n* = 48), and maternal education (*n* = 48). All the included studies in our meta-analysis reached a high NOS score (> 5) (***Supplementary Table 1***), indicating that the quality of the research methods employed was sufficient to ensure reliable estimates of the associations between PM_2.5_/PM_10_ exposure throughout gestation and birth weight.

### Effects of air pollutants on birth weight

#### PM_2.5_ exposure on birth weight and subgroup analyses

***Supplementary Fig. 1*** (available online) presents the overall pooled birth weight estimations. The pooled *β* for birth weight was −10.69 (95% CI: −16.97–−4.42) per 10 μg/m^3^ increase in PM_2.5_ levels (***Supplementary Fig. 1***). The outcomes from the subgroup analyses according to exposure assessment methods were consistent with the overall findings, except for those using AOD (***Supplementary Fig. 2***, available online), with *β* being 13.64 (95% CI: 10.17–17.12). We stratified participants into subgroups based on maternal age (< 30 *vs.* ≥ 30 years) and found that among mothers aged < 30 years, each 10 μg/m³ increase in PM_2.5_ exposure was associated with a *β* coefficient of 0.19 (95% CI: −4.40–4.78) for birth weight, though this association was not statistically significant. For the maternal age ≥30 group, the association was statistically significant and consistent with the overall trend (***Supplementary Fig. 3***, available online). Subgroup analysis based on exposure window indicated that, except for the first trimester (with *β* being 2.55 [95% CI: −11.94–17.04]), the results of the other subgroups mirrored the overall results. Analyses across all three trimesters produced similar results, indicating no significant differences between subgroups (***Supplementary Fig. 4***, available online).

**Figure 2:**
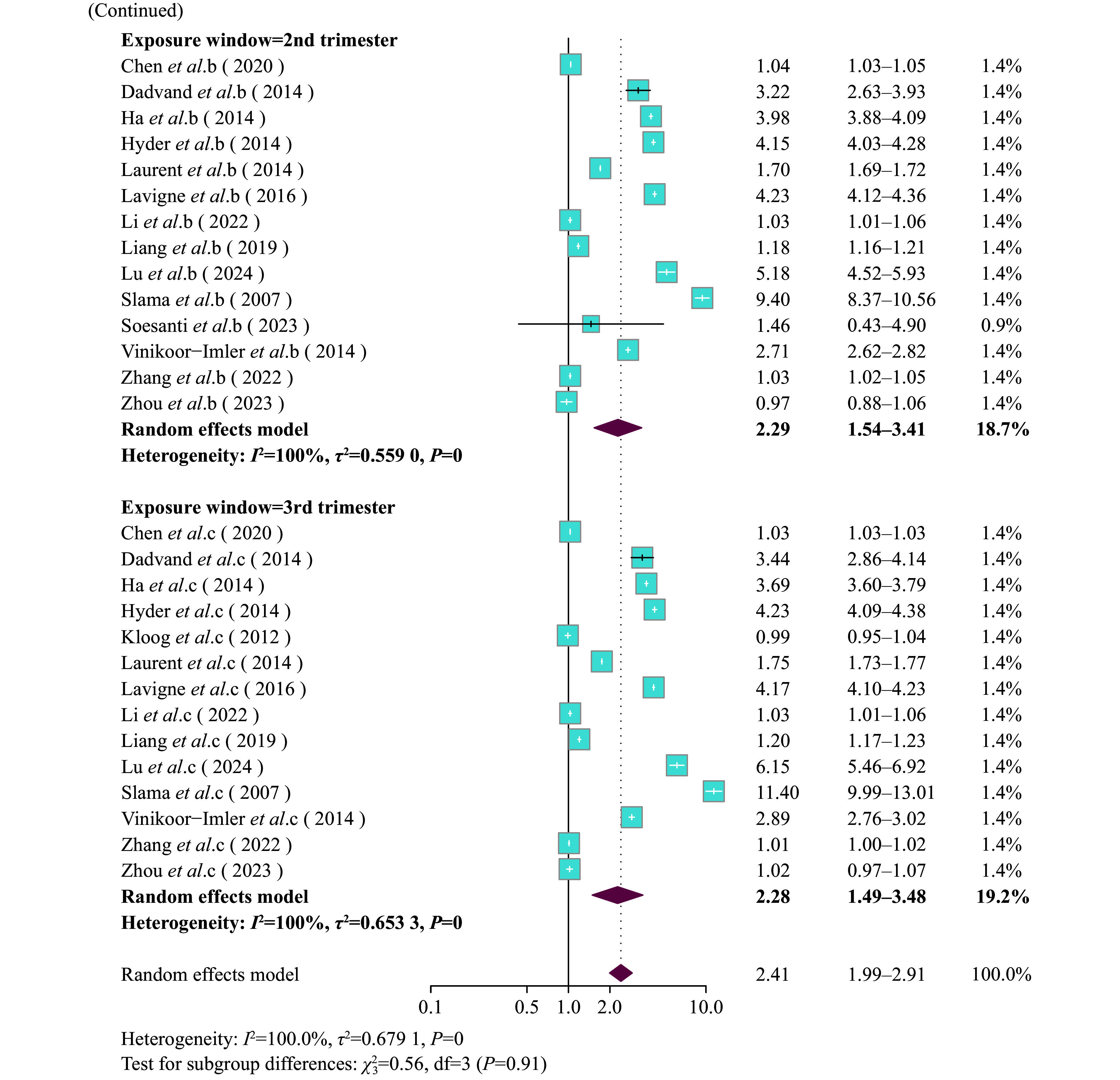
Forest plot of pooled effect estimates (odds ratio [OR] with 95% confidence interval [CI]) for the risk of LBW associated with PM_2.5_ exposure (per 10 μg/m^3^ increment) during the entire pregnancy and three trimesters, stratified by gestational exposure window, analyzed using the random effects model with the subgroup specified as pregnancy exposure window. a: first trimester, b: second trimester, c: third trimester, AP: entire pregnancy.

A positive association between PM_2.5_ exposure throughout gestation and the LBW risk was observed (***Supplementary Fig. 5***, available online). For every 10 μg/m^3^ increase in PM_2.5_ levels, the risk of LBW significantly increased (OR = 2.41, 95% CI: 1.99–2.91). The subgroup analysis by trimester indicated a significant association between PM_2.5_ exposure and the risk of LBW across different trimesters (entire pregnancy: OR = 2.61, 95% CI: 1.91–3.57; T1: OR = 2.19, 95% CI: 1.44–3.33; T2: OR = 2.29, 95% CI: 1.54–3.41; and T3: OR = 2.28, 95% CI: 1.49–3.48) (***Fig. 2***). The subgroup analysis by maternal age indicated that the impact of PM_2.5_ exposure on LBW was particularly stronger in mothers aged 30 and above (OR = 3.69, 95% CI: 2.81–4.84) compared with mothers under 30 years old (OR = 1.61, 95% CI: 1.28–2.04) (***Fig. 3***). Additionally, the subgroup analysis based on exposure assessment methods demonstrated that different exposure assessment methods significantly influenced the estimated impacts of exposure to PM_2.5_ throughout gestation on LBW. The ORs of LBW were as follows: monitor subgroup (OR = 1.05, 95% CI: 1.02–1.09), IDW subgroup (OR = 3.49, 95% CI: 0.46–26.49), LUR subgroup (OR = 3.33, 95% CI: 2.26–4.90), AOD subgroup (OR = 1.04, 95% CI: 1.03–1.05), and other methods subgroup (OR = 3.40, 95% CI: 2.74–4.22) (***Supplementary Fig. 6***, available online).

**Figure 2:**
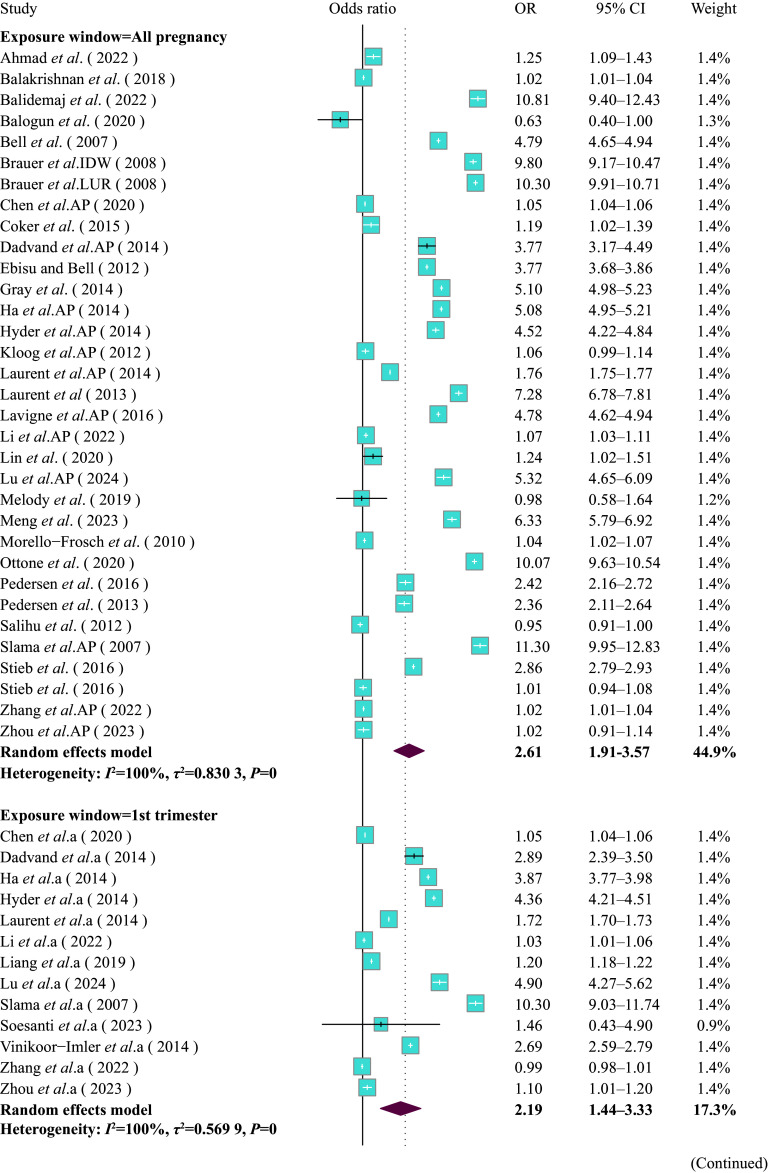


**Figure 3:**
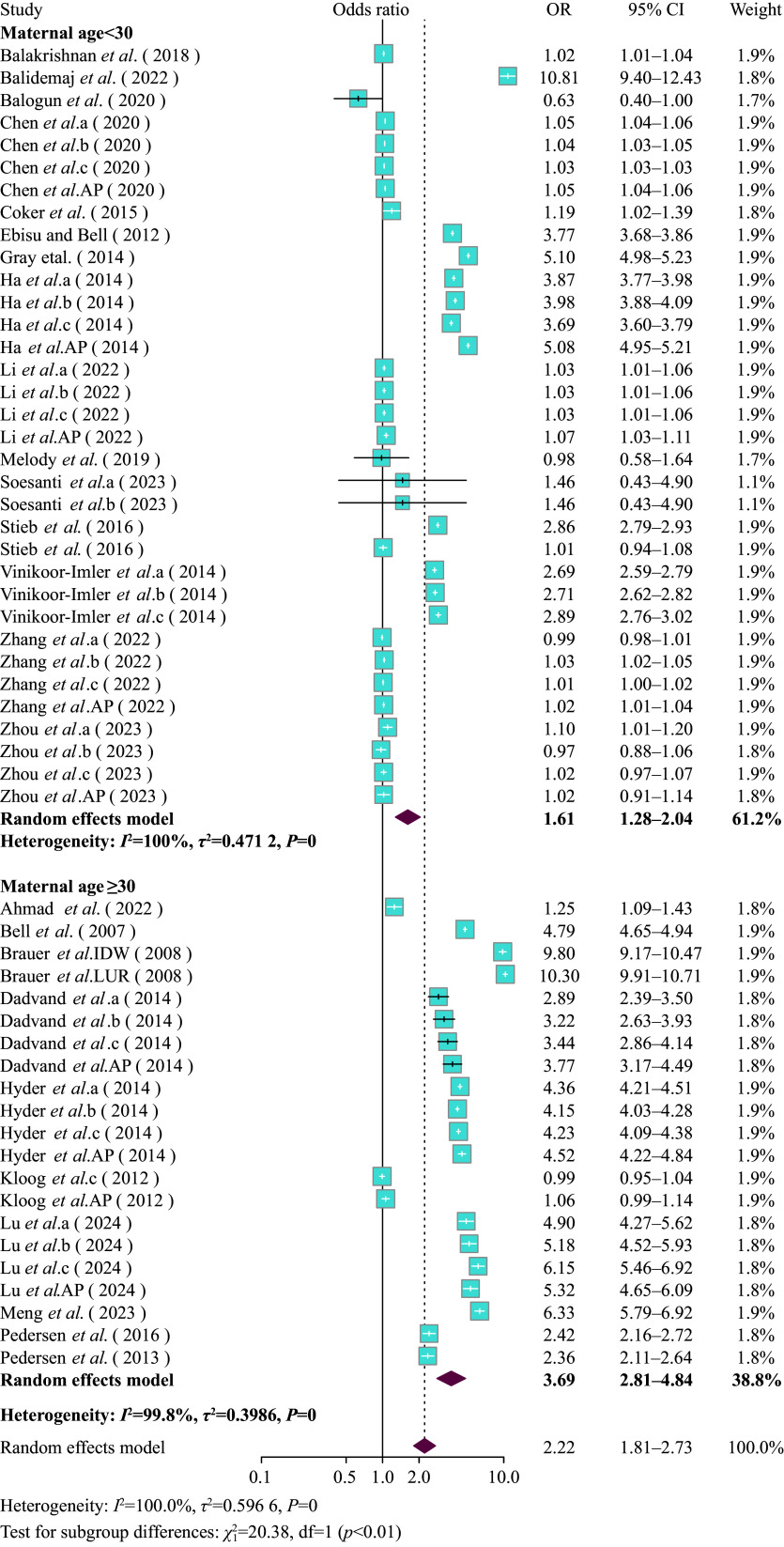
Forest plot of pooled effect estimates (odds ratio [OR] with 95% confidence interval [CI]) for the risk of LBW associated with PM_2.5_ exposure (per 10 μg/m^3^ increment) during pregnancy, stratified by maternal age, analyzed using the random effects model with the subgroup specified as maternal age. a: first trimester, b: second trimester, c: third trimester, AP: entire pregnancy.

#### PM_10_ exposure on birth weight and subgroup analyses

For associations between PM_10_ exposure and birth weight, every 10 μg/m^3^ increase in PM_10_ levels resulted in a pooled *β* of −3.34 (95% CI: −9.01–2.33) (***Supplementary Fig. 7***, available online). Subgroup analyses based on exposure assessment methods indicated that no strong negative correlation was observed between exposure to PM_10_ and the decreased birth weight across methods used (***Supplementary Fig. 8***, available online). Additionally, no significant differences were observed between the two age groups (***Supplementary Fig. 9***, available online). The subgroup analysis based on different trimesters also yielded similar conclusions (***Supplementary Fig. 10***, available online).

For the association between PM_10_ and risk of LBW, a 10 μg/m^3^ increase in the mass concentration of PM_10_ was associated with the risk of LBW (OR = 1.46, 95% CI: 1.16–1.84) (***Fig. 4***). Results were significant in the IDW, LUR, and other groups (IDW group: OR = 3.24, 95% CI: 1.61–6.52; LUR group: OR = 2.06, 95% CI: 1.61–2.65; other group: OR = 1.48, 95% CI: 1.10–2.00) (***Supplementary Fig. 11***, available online). The subgroup analysis by maternal age showed that the influence of PM_10_ exposure on LBW was significant in the maternal age ≥ 30 group (OR = 2.08, 95% CI: 1.34–3.25) (***Supplementary Fig. 12***, available online). The subgroup analysis based on exposure assessment indicated that different exposure assessment methods had a significant impact on the estimated value of the influence of PM_10_ exposure on LBW (***Supplementary Fig. 11***). The subgroup analysis by pregnancy exposure window revealed that PM_10_ exposure was significantly associated with LBW risk throughout the whole pregnancy (OR = 1.80, 95% CI: 1.17–2.77) (***Supplementary Fig. 13***, available online).

**Figure 4:**
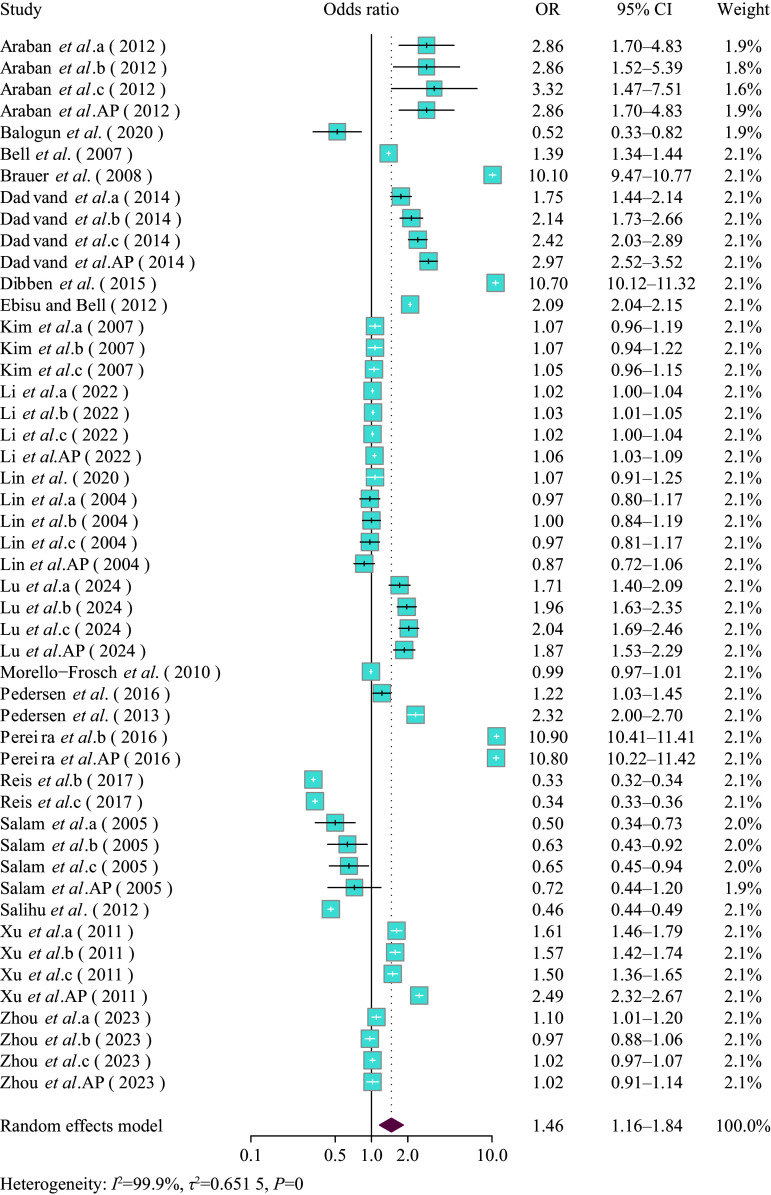
Forest plot of pooled effect estimates (odds ratio [OR] with 95% confidence interval [CI]) for the risk of LBW associated with PM_10_ exposure (per 10 μg/m^3^ increment) during pregnancy, analyzed using the random effects model. a: first trimester, b: second trimester, c: third trimester, AP: entire pregnancy.

### Sensitivity analyses and publication bias

We conducted sensitivity analyses to assess the robustness of the results. Given that 10 studies included in the overall meta-analysis were not analyzed in subgroup analysis by maternal age due to missing or ambiguous information, sensitivity analyses on maternal age were additionally performed. The sensitivity analysis indicated that none of the included studies affected the robustness of association between PM_2.5_ exposure and birth weight (***Supplementary Fig. 14***, available online) or LBW (***Supplementary Fig. 15***, available online). For the association between PM_10_ exposure during gestation and birth weight, the results were consistent after excluding individual studies (***Supplementary Fig. 16***, available online). For the association between PM_10_ exposure throughout gestation and LBW, the pooled effect estimates and statistical significance were not significantly altered by the exclusion of any single study separately (***Supplementary Fig. 17***, available online). These results reinforce the conclusion of a robust negative association between maternal PM_2.5_/PM_10_ exposure throughout gestation and the outcome of LBW. Moreover, our findings support the hypothesis that maternal exposure to PM_2.5_ during pregnancy may lead to lower birth weights in infants.

The funnel plots depicting the general pooled estimated outcomes appeared roughly symmetrical (***Supplementary Fig. 18***, available online), indicating no significant publication bias. However, Egger's test revealed publication bias in the analyses focused on PM_2.5_ and birth weight. In the overall meta-analyses concerning the correlation between PM_2.5_/PM_10_ and LBW, Thompson's test revealed no significant publication bias (***Supplementary Table 2***, available online).

## Discussion

Our meta-analysis revealed a clear association between maternal exposure to PM_2.5_ and PM_10_ throughout gestation and an increased risk of LBW. Subgroup analysis indicated that the effects of PM_2.5_ and PM_10_ exposure on LBW were more pronounced in women aged ≥ 30 years. In addition, the effect of PM_2.5_ on LBW was significant across all trimesters. The results were consistent with previous studies^[21–24]^, which also suggested that maternal exposure to PM_2.5_ and PM_10_ increases the risk of LBW. LBW infants have higher mortality rates and slower physical and neural development, compared with normal birth weight infants^[25]^. All pregnant women are exposed to air pollution to some extent, and fetuses are particularly vulnerable due to their critical periods of organogenesis. Therefore, it is crucial to improve fetal health by reducing exposure to particulate matter such as PM_2.5_ and PM_10_.

We also investigated the effects of PM exposure during different gestational windows on birth weight and the risk of LBW to identify critical exposure periods. Our findings indicated that pregnancy exposure to PM_2.5_ and PM_10_ was associated with an increased risk of LBW in the subgroup analysis. However, since the OR values across the three exposure windows were very close and their 95% CIs nearly completely overlapped, we were unable to identify a specific trimester with a stronger association. Furthermore, the associations between exposures to PM_2.5_ and PM_10_ across different exposure windows and the reduction in birth weight were not statistically significant, thereby preventing us from reaching a definite conclusion. Excessive PM_2.5_ exposure during pregnancy may induce DNA damage through oxidative stress^[26]^ or trigger intrauterine inflammation^[27]^, which is likely to affect the placenta's delivery of oxygen and nutrients to the fetus, resulting in lower birth weight.

In addition, we examined the impact of maternal age during pregnancy. Given the declining birth rate in China and the rising maternal age, it is of great importance to explore the effects of pollutant exposure on birth outcomes in older pregnant women. Therefore, we divided the discussion based on a maternal age cutoff of 30 years. We found that pregnant women aged 30 and older were significantly more likely to deliver LBW infants due to PM_2.5_ exposure, compared with younger mothers. This finding suggests that all pregnant women, particularly those over 30, should be more mindful of PM_2.5_ levels in their environment. Our findings are consistent with previous research^[28]^. Older pregnant women are more likely to experience complications throughout gestation, such as preeclampsia and gestational hypertension^[29]^, which can lead to adverse outcomes such as LBW. Moreover, placental function in older women may be less efficient, reducing nutrient supply to the fetus and increasing the risk of fetal growth restriction and LBW. Therefore, older pregnant women should be especially cautious about particulate matter exposure.

We also conducted subgroup analyses to determine whether LBW risk varied across different exposure assessment methods. Our findings indicated that PM_2.5_ exposure was associated with reduced birth weight, except in the AOD subgroup. This inconsistency may be attributed to the limited number of studies using AOD, making the conclusion tentative and warranting further investigation. Regardless of whether the exposure was to PM_2.5_ or PM_10_, the associations between different exposure assessment methods and LBW risks were inconsistent. Our comparative analysis of air pollution exposure assessment models revealed distinct methodological characteristics and application limitations. The AOD model, based on satellite remote sensing, is useful in large-scale studies but constrained by its 10 km spatial resolution, which necessitates integration with ground monitoring to achieve < 1 km precision. The LUR model offers finer spatial resolution by incorporating multiple geographic variables, but requires intensive data collection and is influenced by the spatial distribution of monitoring stations. The IDW model, which assigns weights based on distance, is suitable for small-scale studies but becomes less accurate when monitoring stations are sparse. The uneven distribution or limited coverage of monitoring sites may introduce bias. Based on our subgroup findings and cost-benefit perspective, we suggest that the LUR model is preferable. We also recommend the use of personal monitoring devices to assess individual pollutant exposure levels, as applied in several recent studies^[30]^. This approach may help reduce heterogeneity introduced by differing assessment methods.

In the present study, we explored the adverse influence of both PM_2.5_ and PM_10_ on birth weight abnormalities to provide a more comprehensive meta-analysis. However, most of the overall studies and subgroup studies showed heterogeneity, suggesting that important confounders, such as regional differences, parental education, and individual-level exposure data, were not accounted for in the included studies. Additionally, many subgroup analyses were limited by a small number of studies, indicating the need for future studies to confirm our conclusions.

In conclusion, this study provided a meta-analysis and systematic evaluation of the epidemiological data on the impacts of PM_2.5_ and PM_10_ exposure during pregnancy on birth weight. Maternal exposure to PM_2.5_ and PM_10_ was associated with an increased risk of LBW across all trimesters, with a greater risk observed in mothers aged 30 and older. Future studies should focus on refining exposure assessment methods, identifying sensitive exposure windows, examining the effects of maternal age, and evaluating regional differences in pollutant concentrations to deepen our understanding of the mechanisms through which air pollution affects birth outcomes.

## SUPPLEMENTARY DATA

Supplementary data to this article can be found online.
