# Supplementary material for: Author correction: Association between exposure to particulate matter during pregnancy and birthweight: A systematic review and a meta-analysis of birth cohort studies
Source: J Biomed Res. 2025 Jul 16;39(5):538–48. doi: 10.7555/JBR.38.20240383 (PMC12481676; doi:10.7555/JBR.38.20240383)
Supplement: Supplementary file 1 — Supplementary data to this article can be found online. [file jbr-39-5-538-Supplementary.pdf]

Available online at www.jbr-pub.org.cn
Open Access at PubMed Central
JBR
Journal of Biomedical Research, 2025 39(5): 1–19

Supplementary Data

Author correction: Association between exposure to particulate matter during pregnancy and birthweight: A systematic review and a meta-analysis of birth cohort studies

Yinwen Ji[1,2,3], Fei Song[4], Bo Xu[1,2], Yining Zhu[5], Chuncheng Lu[1,2], Yankai Xia[1,2,✉]

[1]*State Key Laboratory of Reproductive Medicine, Institute of Toxicology, Nanjing Medical University, Nanjing, Jiangsu 211166, China;*
[2]*Key Laboratory of Modern Toxicology of Ministry of Education, School of Public Health, Nanjing Medical University, Nanjing, Jiangsu 211166, China;*
[3]*Department of Research and Education, The Children's Hospital, Zhejiang University School of Medicine, Hangzhou, Zhejiang 310003, China;*
[4]*Department of Epidemiology and Biostatistics, School of Public Health, Tianjin Medical University, Tianjin 300070, China;*
[5]*Department of Thoracic Surgery, The First School of Clinical Medicine, Nanjing Medical University, Nanjing, Jiangsu 210029, China.*

Supplementary Table 1 (available online) Characteristics of the studies included in the meta-analysis.
Supplementary Table 2 (available online) Publication bias analyses of the meta-analyses to assess changes in birth weight or low birth weight associated with maternal exposure to $PM_{2.5}$ or PM_{10}.

✉Corresponding author: Yankai Xia, State Key Laboratory of Reproductive Medicine and Offspring Health, School of Public Health, Nanjing Medical University, No.101 Longmian Avenue, Nanjing, Jiangsu 211166, China. E-mail: yankaixia@njmu.edu.cn.
Received: 08 November 2024; Revised: 05 July 2025; Accepted: 07 July 2025; Published online: 16 July 2025

CLC number: R715.3, Document code: A
The authors reported no conflict of interests.
This is an open access article under the Creative Commons Attribution (CC BY 4.0) license, which permits others to distribute, remix, adapt and build upon this work, for commercial use, provided the original work is properly cited.

© 2025 by Journal of Biomedical Research.
https://doi.org/10.7555/JBR.38.20240383

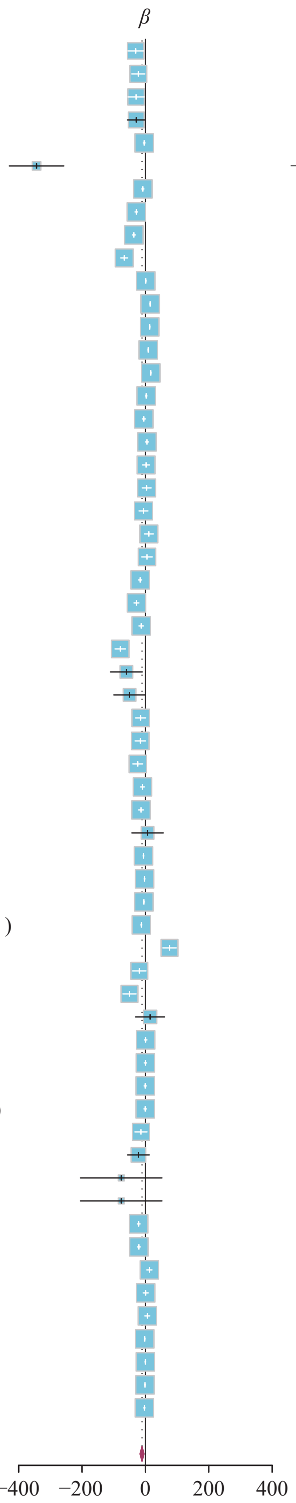

Study	β	95% CI	Weight
Bai *et al.*a (2022)	−30.53	−55.15–−5.91	1.5%
Bai *et al.*b (2022)	−22.32	−42.82–−1.82	1.6%
Bai *et al.*c (2022)	−29.47	−54.99–−3.95	1.5%
Bai *et al.*AP (2022)	−28.92	−56.95–−0.89	1.4%
Balakrishnan *et al.* (2018)	−3.92	−6.76–−1.08	1.9%
Balidemaj *et al.* (2022)	−343.43	−429.30–−257.56	0.4%
Basu *et al.* (2014)	−7.93	−11.24–−4.63	1.9%
Bell *et al.*b (2007)	−28.64	−32.73–−24.55	1.9%
Bell *et al.*c (2007)	−36.36	−40.91–−31.82	1.9%
Bell *et al.*AP (2007)	−66.82	−77.73–−55.91	1.8%
Dong *et al.* (2020)	1.23	−0.00–2.46	1.9%
Du *et al.*a (2022)	14.93	13.97–15.89	1.9%
Du *et al.*b (2022)	13.75	12.81–14.69	1.9%
Du *et al.*c (2022)	8.79	8.08–9.50	1.9%
Du *et al.*AP (2022)	17.15	16.13–18.17	1.9%
Han *et al.*a (2018)	2.38	−0.23–4.99	1.9%
Han *et al.*b (2018)	−4.94	−9.84–−0.04	1.9%
Han *et al.*c (2018)	4.96	0.00–9.92	1.9%
Han *et al.*AP (2018)	2.14	−9.03–13.31	1.8%
Hannam *et al.*a (2014)	4.00	−10.00–18.00	1.8%
Hannam *et al.*b (2014)	−6.00	−20.00–8.00	1.8%
Hannam *et al.*c (2014)	11.00	−3.00–25.00	1.8%
Hannam *et al.*AP (2014)	5.00	−11.50–21.50	1.7%
Hyder *et al.*a (2014)	−16.60	−21.58–−11.62	1.9%
Hyder *et al.*b (2014)	−28.22	−35.89–−20.54	1.9%
Hyder *et al.*c (2014)	−13.28	−20.33–−6.22	1.9%
Hyder *et al.*AP (2014)	−79.25	−95.85–−62.66	1.7%
Johnson *et al.*b (2022)	−60.00	−110.00–−10.00	0.9%
Johnson *et al.*c (2022)	−50.00	−99.51–−0.49	0.9%
Keller *et al.*a (2017)	−15.00	−30.99–0.99	1.7%
Keller *et al.*b (2017)	−16.00	−32.50–0.50	1.7%
Keller *et al.*c (2017)	−24.00	−39.50–−8.50	1.7%
Kloog *et al.*c (2012)	−9.20	−15.05–−3.35	1.9%
Kloog *et al.*AP (2012)	−13.80	−21.32–−6.28	1.9%
Melody *et al.* (2019)	6.81	−42.58–56.20	0.9%
Morello−Frosch *et al.*a (2010)	−6.00	−7.25–−4.75	1.9%
Morello−Frosch *et al.*b (2010)	−2.60	−3.95–−1.25	1.9%
Morello−Frosch *et al.*c (2010)	−4.70	−5.95–−3.45	1.9%
Morello−Frosch *et al.*AP (2010)	−12.80	−14.30–−11.30	1.9%
Ouidir *et al.*a (2021)	76.20	55.23–97.17	1.6%
Ouidir *et al.*b (2021)	−19.20	−40.15–1.75	1.6%
Ouidir *et al.*c (2021)	−50.20	−70.84–−29.56	1.6%
Ouidir *et al.*AP (2021)	14.80	−30.91–60.51	1.0%
Payne−Sturges *et al.*a (2022)	0.80	−3.20–4.80	1.9%
Payne−Sturges *et al.*b (2022)	−0.04	−3.78–3.70	1.9%
Payne−Sturges *et al.*c (2022)	−0.70	−3.99–2.59	1.9%
Payne−Sturges *et al.*AP (2022)	−0.70	−4.54–3.14	1.9%
Pedersen *et al.* (2013)	−14.00	−33.00–5.00	1.6%
Schembari *et al.* (2015)	−22.00	−56.01–12.01	1.2%
Soesanti *et al.*a (2023)	−76.25	−204.69–52.19	0.2%
Soesanti *et al.*b (2023)	−76.25	−204.69–52.19	0.2%
Stieb *et al.* (2016)	−21.14	−25.43–−16.86	1.9%
Stieb *et al.* (2016)	−20.50	−24.65–−16.35	1.9%
Vinikoor−Imler *et al.*a (2014)	12.66	4.59–20.73	1.9%
Vinikoor−Imler *et al.*b (2014)	0.94	−7.00–8.89	1.9%
Vinikoor−Imler *et al.*c (2014)	6.11	−1.64–13.87	1.9%
Wu *et al.*a (2021)	−2.01	−3.09–−0.93	1.9%
Wu *et al.*b (2021)	−0.18	−1.40–1.04	1.9%
Wu *et al.*c (2021)	−1.31	−2.37–−0.25	1.9%
Wu *et al.*AP (2021)	−3.21	−5.27–−1.15	1.9%
Random effects model	−10.69	−16.97–−4.42	100.0%

Heterogeneity: I^2=98.7%, τ^2=533.667 2, P=0

Supplementary Fig. 1 **Forest plot of pooled estimates of changes (regression coefficients [*β*] with 95% confidence interval [CI]) in birth weight associated with $PM_{2.5}$ exposure during the entire pregnancy, analyzed using the random effects model.** a: first trimester, b: second trimester, c: third trimester, AP: entire pregnancy.

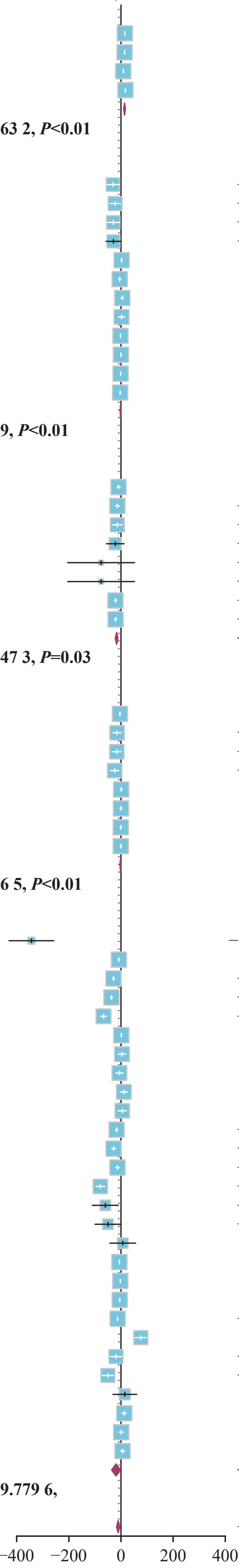

Study	β	95% CI	Weight
**Method=AOD**			
Du *et al.*a (2022)	14.93	13.97–15.89	1.9%
Du *et al.*b (2022)	13.75	12.81–14.69	1.9%
Du *et al.*c (2022)	8.79	8.08–9.50	1.9%
Du *et al.*AP (2022)	17.15	16.13–18.17	1.9%
**Random effects model**	**13.64**	**10.17–17.12**	**7.7%**
**Heterogeneity: I^2=98.6%, τ^2=12.363 2, *P*<0.01**			
**Method=IDW**			
Bai *et al.*a (2022)	−30.53	−55.15–−5.91	1.5%
Bai *et al.*b (2022)	−22.32	−42.82–−1.82	1.6%
Bai *et al.*c (2022)	−29.47	−54.99–−3.95	1.5%
Bai *et al.*AP (2022)	−28.92	−56.95–−0.89	1.4%
Han *et al.*a (2018)	2.38	−0.23–4.99	1.9%
Han *et al.*b (2018)	−4.94	−9.84–−0.04	1.9%
Han *et al.*c (2018)	4.96	0.00–9.92	1.9%
Han *et al.*AP (2018)	2.14	−9.03–13.31	1.8%
Wu *et al.*a (2021)	−2.01	−3.09–−0.93	1.9%
Wu *et al.*b (2021)	−0.18	−1.40–1.04	1.9%
Wu *et al.*c (2021)	−1.31	−2.37–−0.25	1.9%
Wu *et al.*AP (2021)	−3.21	−5.27–−1.15	1.9%
**Random effects model**	**−1.37**	**−3.32–0.58**	**21.1%**
**Heterogeneity: I^2=74%, τ^2=5.604 9, *P*<0.01**			
**Method=LUR**			
Kloog *et al.*c (2012)	−9.20	−15.05–−3.35	1.9%
Kloog *et al.*AP (2012)	−13.80	−21.32–−6.28	1.9%
Pedersen *et al.* (2013)	−14.00	−33.00–5.00	1.6%
Schembari *et al.* (2015)	−22.00	−56.01–12.01	1.2%
Soesanti *et al.*a (2023)	−76.25	−204.69–52.19	0.2%
Soesanti *et al.*b (2023)	−76.25	−204.69–52.19	0.2%
Stieb *et al.* (2016)	−21.14	−25.43–−16.86	1.9%
Stieb *et al.* (2016)	−20.50	−24.65–−16.35	1.9%
**Random effects model**	**−16.76**	**−21.86–−11.66**	**10.8%**
**Heterogeneity: I^2=53.8%, τ^2=21.847 3, *P*=0.03**			
**Method=monitor**			
Balakrishnan *et al.* (2018)	−3.92	−6.76–−1.08	1.9%
Keller *et al.*a (2017)	−15.00	−30.99–0.99	1.7%
Keller *et al.*b (2017)	−16.00	−32.50–0.50	1.7%
Keller *et al.*c (2017)	−24.00	−39.50–−8.50	1.7%
Payne−Sturges *et al.*a (2022)	0.80	−3.20–4.80	1.9%
Payne−Sturges *et al.*b (2022)	−0.04	−3.78–3.70	1.9%
Payne−Sturges *et al.*c (2022)	−0.70	−3.99–2.59	1.9%
Payne−Sturges *et al.*AP (2022)	−0.70	−4.54–3.14	1.9%
**Random effects model**	**−1.92**	**−3.92–0.08**	**14.7%**
**Heterogeneity: I^2=62.6%, τ^2=2.236 5, *P*<0.01**			
**Method=others**			
Balidemaj *et al.* (2022)	−343.43	−429.30–−257.56	0.4%
Basu *et al.* (2014)	−7.93	−11.24–−4.63	1.9%
Bell *et al.*b (2007)	−28.64	−32.73–−24.55	1.9%
Bell *et al.*c (2007)	−36.36	−40.91–−31.82	1.9%
Bell *et al.*AP (2007)	−66.82	−77.73–−55.91	1.8%
Dong *et al.* (2020)	1.23	−0.00–2.46	1.9%
Hannam *et al.*a (2014)	4.00	−10.00–18.00	1.8%
Hannam *et al.*b (2014)	−6.00	−20.00–8.00	1.8%
Hannam *et al.*c (2014)	11.00	−3.00–25.00	1.8%
Hannam *et al.*AP (2014)	5.00	−11.50–21.50	1.7%
Hyder *et al.*a (2014)	−16.60	−21.58–−11.62	1.9%
Hyder *et al.*b (2014)	−28.22	−35.89–−20.54	1.9%
Hyder *et al.*c (2014)	−13.28	−20.33–−6.22	1.9%
Hyder *et al.*AP (2014)	−79.25	−95.85–−62.66	1.7%
Johnson *et al.*b (2022)	−60.00	−110.00–−10.00	0.9%
Johnson *et al.*c (2022)	−50.00	−99.51–−0.49	0.9%
Melody *et al.* (2019)	6.81	−42.58–56.20	0.9%
Morello−Frosch *et al.*a (2010)	−6.00	−7.25–−4.75	1.9%
Morello−Frosch *et al.*b (2010)	−2.60	−3.95–−1.25	1.9%
Morello−Frosch *et al.*c (2010)	−4.70	−5.95–−3.45	1.9%
Morello−Frosch *et al.*AP (2010)	−12.80	−14.30–−11.30	1.9%
Ouidir *et al.*a (2021)	76.20	55.23–97.17	1.6%
Ouidir *et al.*b (2021)	−19.20	−40.15–1.75	1.6%
Ouidir *et al.*c (2021)	−50.20	−70.84–−29.56	1.6%
Ouidir *et al.*AP (2021)	14.80	−30.91–60.51	1.0%
Vinkoor−Imler *et al.*a (2014)	12.66	4.59–20.73	1.9%
Vinkoor−Imler *et al.*b (2014)	0.94	−7.00–8.89	1.9%
Vinkoor−Imler *et al.*c (2014)	6.11	−1.64–13.87	1.9%
**Random effects model**	**−19.06**	**−36.14–−1.98**	**45.7%**
**Heterogeneity: I^2=97.2%, τ^2=1 989.779 6, *P*<0.01**			
Random effects model	−10.69	−16.97–−4.42	100.0%

Heterogeneity: I^2=98.7%, τ^2=533.6672, *P*=0
Test for subgroup differences: χ^2_4=109.77, df=4 (*P*<0.01)

Supplementary Fig. 2 **Forest plot of pooled estimates of changes (regression coefficients [*β*] with 95% confidence interval [CI]) in birth weight associated with $PM_{2.5}$ exposure during the entire pregnancy, stratified by exposure assessment methods subgroup, analyzed using the random effects model with the subgroup specified as exposure assessment methods.** a: first trimester, b: second trimester, c: third trimester, AP: entire pregnancy.

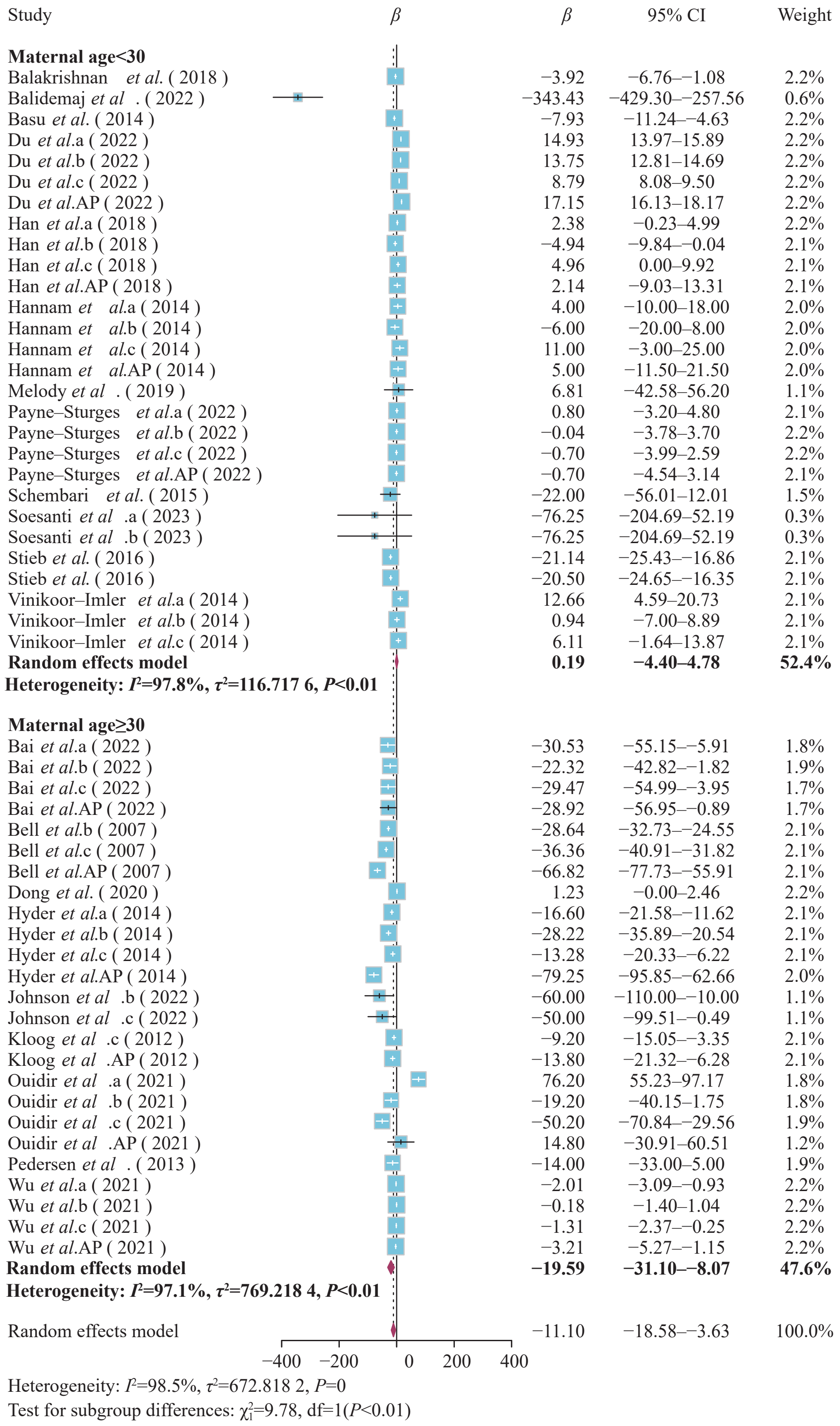

Supplementary Fig. 3 **Forest plot of pooled estimates of changes (regression coefficients [*β*] with 95% confidence interval [CI]) in birth weight associated with $PM_{2.5}$ exposure during the entire pregnancy, stratified by maternal age subgroup, analyzed using the random effects model with the subgroup specified as maternal age.** a: first trimester, b: second trimester, c: third trimester, AP: entire pregnancy.

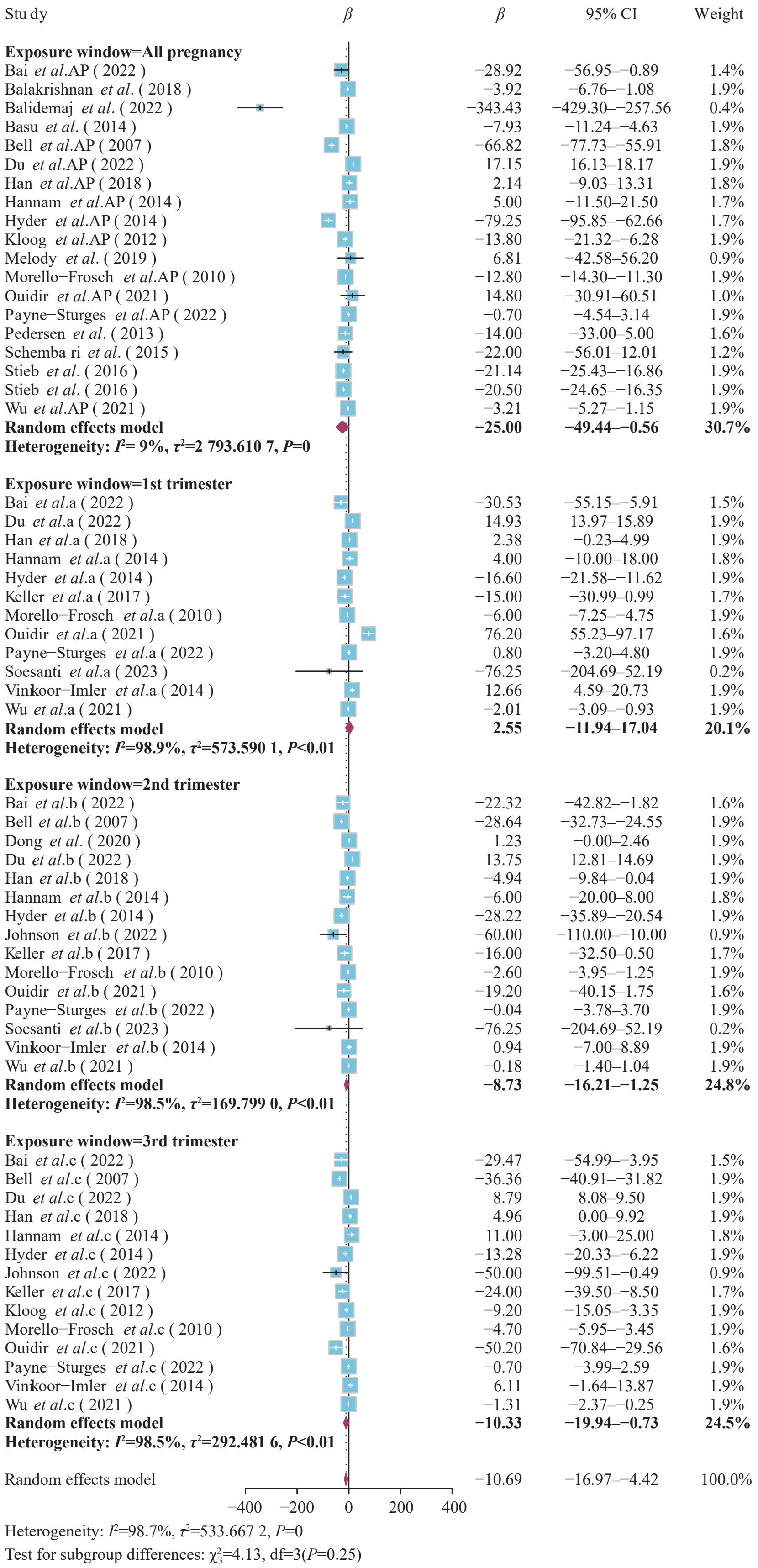

Supplementary Fig. 4 **Forest plot of pooled estimates of changes (regression coefficients [*β*] with 95% confidence interval [CI]) in birth weight associated with $PM_{2.5}$ exposure during the entire pregnancy stratified by pregnancy exposure window subgroup, analyzed using the random effects model with the subgroup specified as pregnancy exposure window.** a: first trimester, b: second trimester, c: third trimester, AP: entire pregnancy.

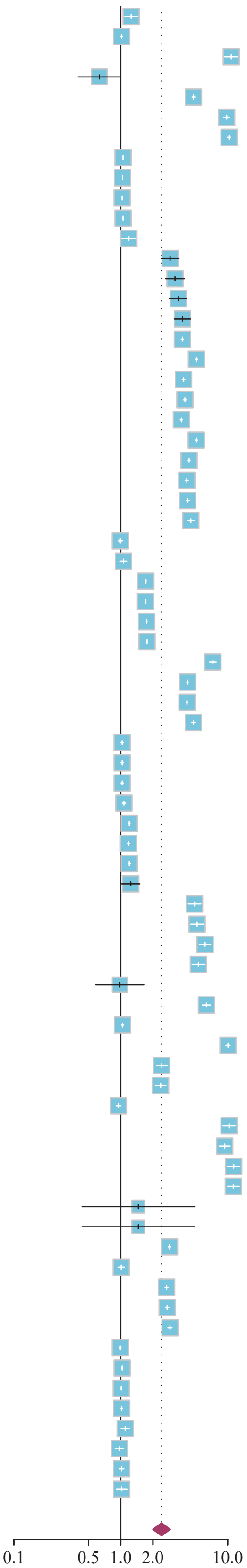

Study	OR	95% CI	Weight
Ahmad *et al.* (2022)	1.25	1.09–1.43	1.4%
Balakrishnan *et al.* (2018)	1.02	1.01–1.04	1.4%
Balidemaj *et al.* (2022)	10.81	9.40–12.43	1.4%
Balogun *et al.* (2020)	0.63	0.40–1.00	1.3%
Bell *et al.* (2007)	4.79	4.65–4.94	1.4%
Brauer *et al.*IDW (2008)	9.80	9.17–10.47	1.4%
Brauer *et al.*LUR (2008)	10.30	9.91–10.71	1.4%
Chen *et al.*a (2020)	1.05	1.04–1.06	1.4%
Chen *et al.*b (2020)	1.04	1.03–1.05	1.4%
Chen *et al.*c (2020)	1.03	1.03–1.03	1.4%
Chen *et al.*AP (2020)	1.05	1.04–1.06	1.4%
Coker *et al.* (2015)	1.19	1.02–1.39	1.4%
Dadvand *et al.*a (2014)	2.89	2.39–3.50	1.4%
Dadvand *et al.*b (2014)	3.22	2.63–3.93	1.4%
Dadvand *et al.*c (2014)	3.44	2.86–4.14	1.4%
Dadvand *et al.*AP (2014)	3.77	3.17–4.49	1.4%
Ebisu and Bell (2012)	3.77	3.68–3.86	1.4%
Gray *et al.* (2014)	5.10	4.98–5.23	1.4%
Ha *et al.*a (2014)	3.87	3.77–3.98	1.4%
Ha *et al.*b (2014)	3.98	3.88–4.09	1.4%
Ha *et al.*c (2014)	3.69	3.60–3.79	1.4%
Ha *et al.*AP (2014)	5.08	4.95–5.21	1.4%
Hyder *et al.*a (2014)	4.36	4.21–4.51	1.4%
Hyder *et al.*b (2014)	4.15	4.03–4.28	1.4%
Hyder *et al.*c (2014)	4.23	4.09–4.38	1.4%
Hyder *et al.*AP (2014)	4.52	4.22–4.84	1.4%
Kloog *et al.*c (2012)	0.99	0.95–1.04	1.4%
Kloog *et al.*AP (2012)	1.06	0.99–1.14	1.4%
Laurent *et al.*a (2014)	1.72	1.70–1.73	1.4%
Laurent *et al.*b (2014)	1.70	1.69–1.72	1.4%
Laurent *et al.*c (2014)	1.75	1.73–1.77	1.4%
Laurent *et al.*AP (2014)	1.76	1.75–1.77	1.4%
Laurent *et al.* (2013)	7.28	6.78–7.81	1.4%
Lavigne *et al.*b (2016)	4.23	4.12–4.36	1.4%
Lavigne *et al.*c (2016)	4.17	4.10–4.23	1.4%
Lavigne *et al.*AP (2016)	4.78	4.62–4.94	1.4%
Li *et al.*a (2022)	1.03	1.01–1.06	1.4%
Li *et al.*b (2022)	1.03	1.01–1.06	1.4%
Li *et al.*c (2022)	1.03	1.01–1.06	1.4%
Li *et al.*AP (2022)	1.07	1.03–1.11	1.4%
Liang *et al.*a (2019)	1.20	1.18–1.22	1.4%
Liang *et al.*b (2019)	1.18	1.16–1.21	1.4%
Liang *et al.*c (2019)	1.20	1.17–1.23	1.4%
Lin *et al.* (2020)	1.24	1.02–1.51	1.4%
Lu *et al.*a (2024)	4.90	4.27–5.62	1.4%
Lu *et al.*b (2024)	5.18	4.52–5.93	1.4%
Lu *et al.*c (2024)	6.15	5.46–6.92	1.4%
Lu *et al.*AP (2024)	5.32	4.65–6.09	1.4%
Melody *et al.* (2019)	0.98	0.58–1.64	1.2%
Meng *et al.* (2023)	6.33	5.79–6.92	1.4%
Morello−Frosch *et al.* (2010)	1.04	1.02–1.07	1.4%
Ottone *et al.* (2020)	10.07	9.63–10.54	1.4%
Pedersen *et al.* (2016)	2.42	2.16–2.72	1.4%
Pedersen *et al.* (2013)	2.36	2.11–2.64	1.4%
Salihu *et al.* (2012)	0.95	0.91–1.00	1.4%
Slama *et al.*a (2007)	10.30	9.03–11.74	1.4%
Slama *et al.*b (2007)	9.40	8.37–10.56	1.4%
Slama *et al.*c (2007)	11.40	9.99–13.01	1.4%
Slama *et al.*AP (2007)	11.30	9.95–12.83	1.4%
Soesanti *et al.*a (2023)	1.46	0.43–4.90	0.9%
Soesanti *et al.*b (2023)	1.46	0.43–4.90	0.9%
Stieb *et al.* (2016)	2.86	2.79–2.93	1.4%
Stieb *et al.* (2016)	1.01	0.94–1.08	1.4%
Vinikoor−Imler *et al.*a (2014)	2.69	2.59–2.79	1.4%
Vinikoor−Imler *et al.*b (2014)	2.71	2.62–2.82	1.4%
Vinikoor−Imler *et al.*c (2014)	2.89	2.76–3.02	1.4%
Zhang *et al.*a (2022)	0.99	0.98–1.01	1.4%
Zhang *et al.*b (2022)	1.03	1.02–1.05	1.4%
Zhang *et al.*c (2022)	1.01	1.00–1.02	1.4%
Zhang *et al.*AP (2022)	1.02	1.01–1.04	1.4%
Zhou *et al.*a (2023)	1.10	1.01–1.20	1.4%
Zhou *et al.*b (2023)	0.97	0.88–1.06	1.4%
Zhou *et al.*c (2023)	1.02	0.97–1.07	1.4%
Zhou *et al.*AP (2023)	1.02	0.91–1.14	1.4%
Random effects model	2.41	1.99–2.91	100.0%

Heterogeneity: I^2=100.0%, τ^2=0.679 1, P=0

Supplementary Fig. 5 **Forest plot of pooled associations between $PM_{2.5}$ exposure during the entire pregnancy and low birth weight risk (odds ratio [OR] with 95% confidence interval [CI]), analyzed using the random effects model.** a: first trimester, b: second trimester, c: third trimester, AP: entire pregnancy.

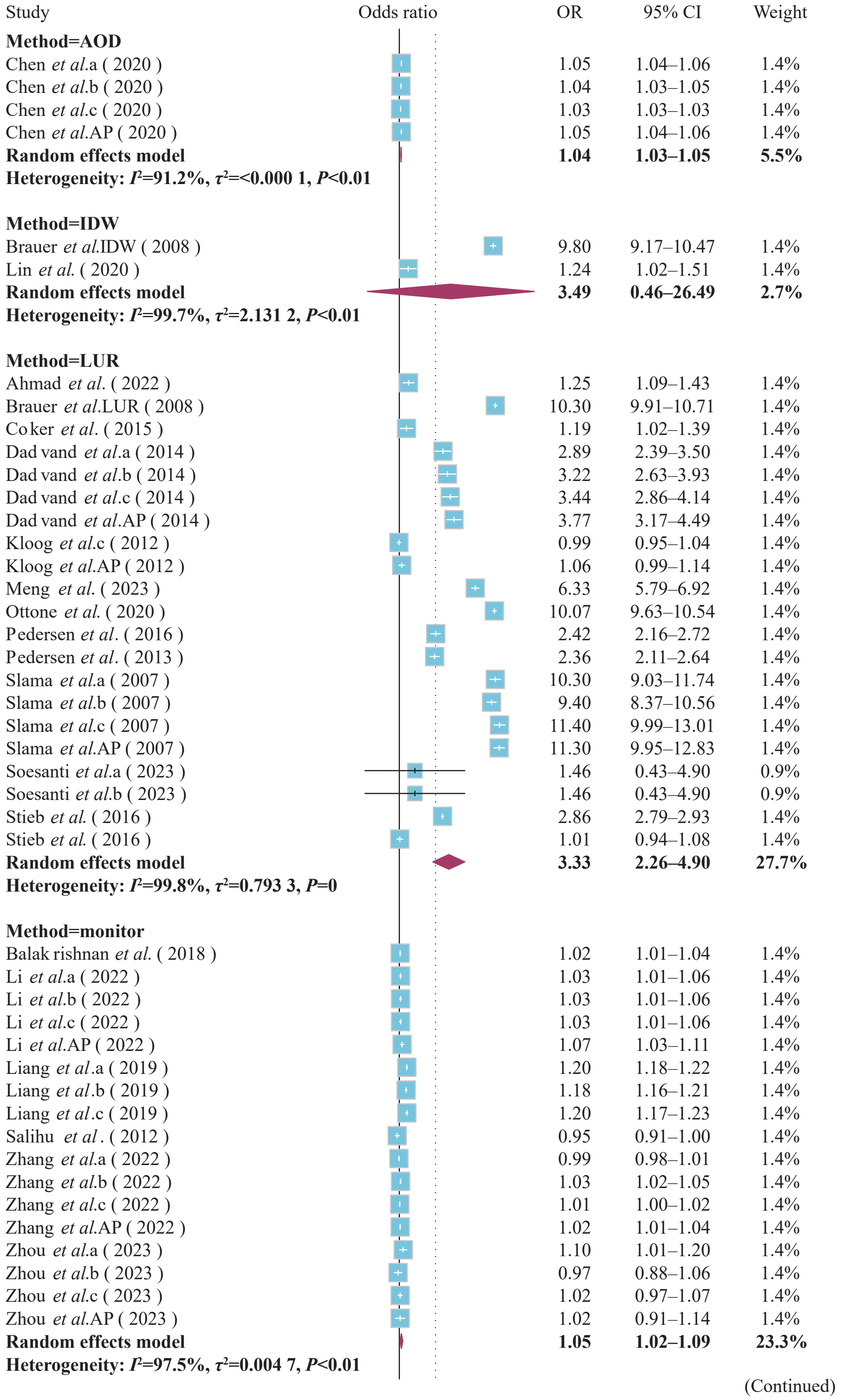
Study
Odds ratio
OR
95% CI
Weight
Method=AOD
Chen et al.a (2020) 1.05 1.04–1.06 1.4%
Chen et al.b (2020) 1.04 1.03–1.05 1.4%
Chen et al.c (2020) 1.03 1.03–1.03 1.4%
Chen et al.AP (2020) 1.05 1.04–1.06 1.4%
Random effects model 1.04 1.03–1.05 5.5%
Heterogeneity: I²=91.2%, τ²=<0.000 1, P<0.01
Method=IDW
Brauer et al.IDW (2008) 9.80 9.17–10.47 1.4%
Lin et al. (2020) 1.24 1.02–1.51 1.4%
Random effects model 3.49 0.46–26.49 2.7%
Heterogeneity: I²=99.7%, τ²=2.131 2, P<0.01
Method=LUR
Ahmad et al. (2022) 1.25 1.09–1.43 1.4%
Brauer et al.LUR (2008) 10.30 9.91–10.71 1.4%
Coker et al. (2015) 1.19 1.02–1.39 1.4%
Dadvand et al.a (2014) 2.89 2.39–3.50 1.4%
Dadvand et al.b (2014) 3.22 2.63–3.93 1.4%
Dadvand et al.c (2014) 3.44 2.86–4.14 1.4%
Dadvand et al.AP (2014) 3.77 3.17–4.49 1.4%
Kloog et al.c (2012) 0.99 0.95–1.04 1.4%
Kloog et al.AP (2012) 1.06 0.99–1.14 1.4%
Meng et al. (2023) 6.33 5.79–6.92 1.4%
Ottone et al. (2020) 10.07 9.63–10.54 1.4%
Pedersen et al. (2016) 2.42 2.16–2.72 1.4%
Pedersen et al. (2013) 2.36 2.11–2.64 1.4%
Slama et al.a (2007) 10.30 9.03–11.74 1.4%
Slama et al.b (2007) 9.40 8.37–10.56 1.4%
Slama et al.c (2007) 11.40 9.99–13.01 1.4%
Slama et al.AP (2007) 11.30 9.95–12.83 1.4%
Soesanti et al.a (2023) 1.46 0.43–4.90 0.9%
Soesanti et al.b (2023) 1.46 0.43–4.90 0.9%
Stieb et al. (2016) 2.86 2.79–2.93 1.4%
Stieb et al. (2016) 1.01 0.94–1.08 1.4%
Random effects model 3.33 2.26–4.90 27.7%
Heterogeneity: I²=99.8%, τ²=0.793 3, P=0
Method=monitor
Balakrishnan et al. (2018) 1.02 1.01–1.04 1.4%
Li et al.a (2022) 1.03 1.01–1.06 1.4%
Li et al.b (2022) 1.03 1.01–1.06 1.4%
Li et al.c (2022) 1.03 1.01–1.06 1.4%
Li et al.AP (2022) 1.07 1.03–1.11 1.4%
Liang et al.a (2019) 1.20 1.18–1.22 1.4%
Liang et al.b (2019) 1.18 1.16–1.21 1.4%
Liang et al.c (2019) 1.20 1.17–1.23 1.4%
Salihu et al. (2012) 0.95 0.91–1.00 1.4%
Zhang et al.a (2022) 0.99 0.98–1.01 1.4%
Zhang et al.b (2022) 1.03 1.02–1.05 1.4%
Zhang et al.c (2022) 1.01 1.00–1.02 1.4%
Zhang et al.AP (2022) 1.02 1.01–1.04 1.4%
Zhou et al.a (2023) 1.10 1.01–1.20 1.4%
Zhou et al.b (2023) 0.97 0.88–1.06 1.4%
Zhou et al.c (2023) 1.02 0.97–1.07 1.4%
Zhou et al.AP (2023) 1.02 0.91–1.14 1.4%
Random effects model 1.05 1.02–1.09 23.3%
Heterogeneity: I²=97.5%, τ²=0.004 7, P<0.01

(Continued)

(Continued)

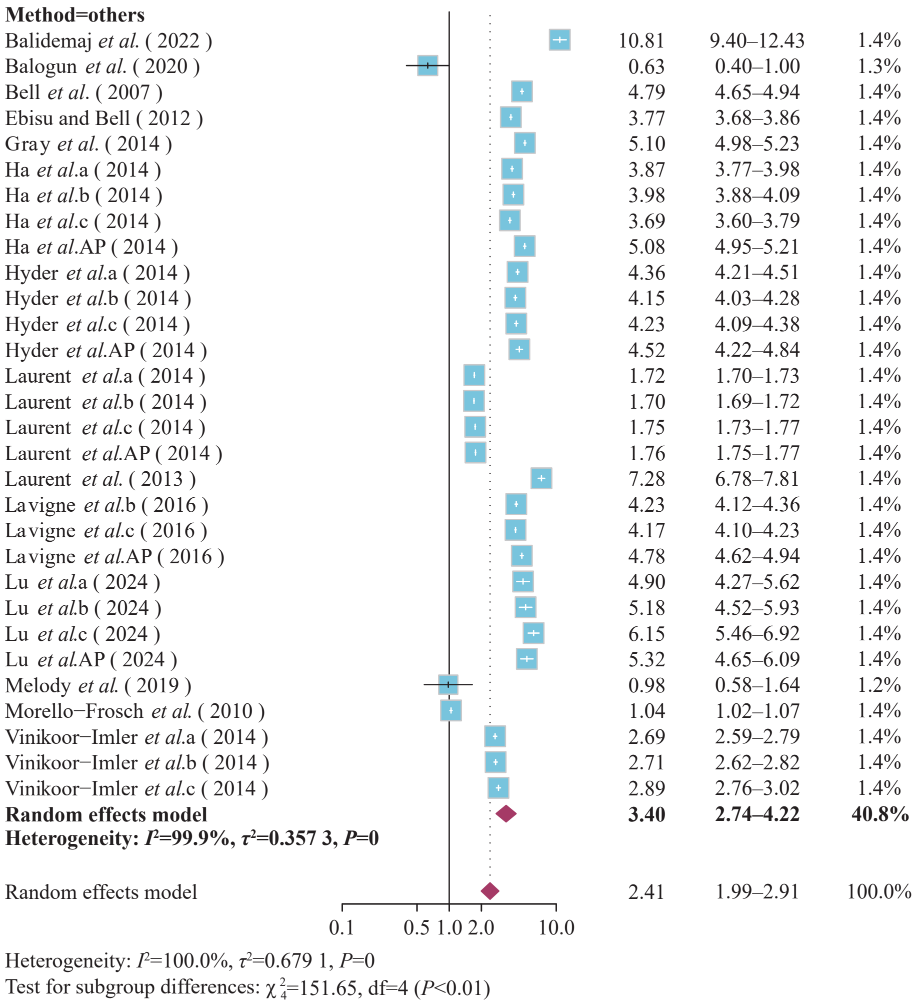

Supplementary Fig. 6 **Forest plot of pooled associations between $PM_{2.5}$ exposure during the entire pregnancy and low birth weight risk (odds ratio [OR] with 95% confidence interval [CI]) in exposure assessment methods subgroups, analyzed using the random effects model with the subgroup specified as exposure assessment methods.** a: first trimester, b: second trimester, c: third trimester, AP: entire pregnancy.

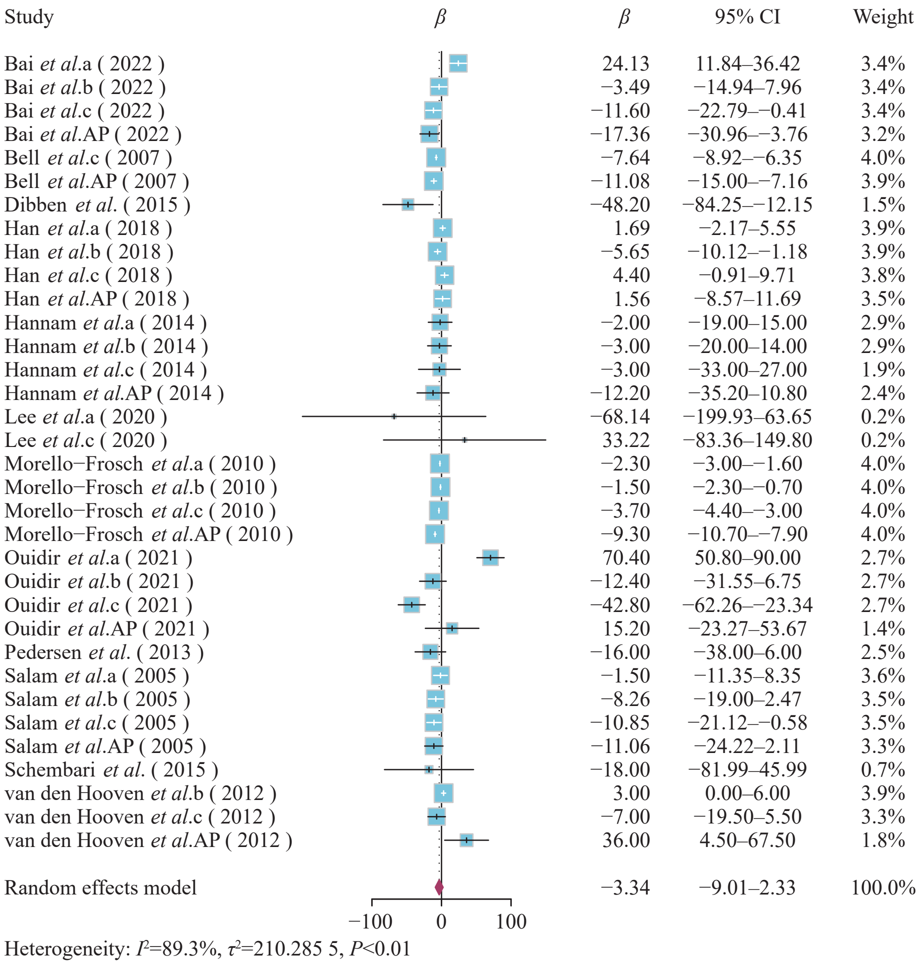

Supplementary Fig. 7 **Forest plot of pooled estimates of changes (regression coefficients [*β*] with 95% confidence interval [CI]) in birth weight associated with PM_{10} exposure during the entire pregnancy, analyzed using the random effects model.** a: first trimester, b: second trimester, c: third trimester, AP: entire pregnancy.

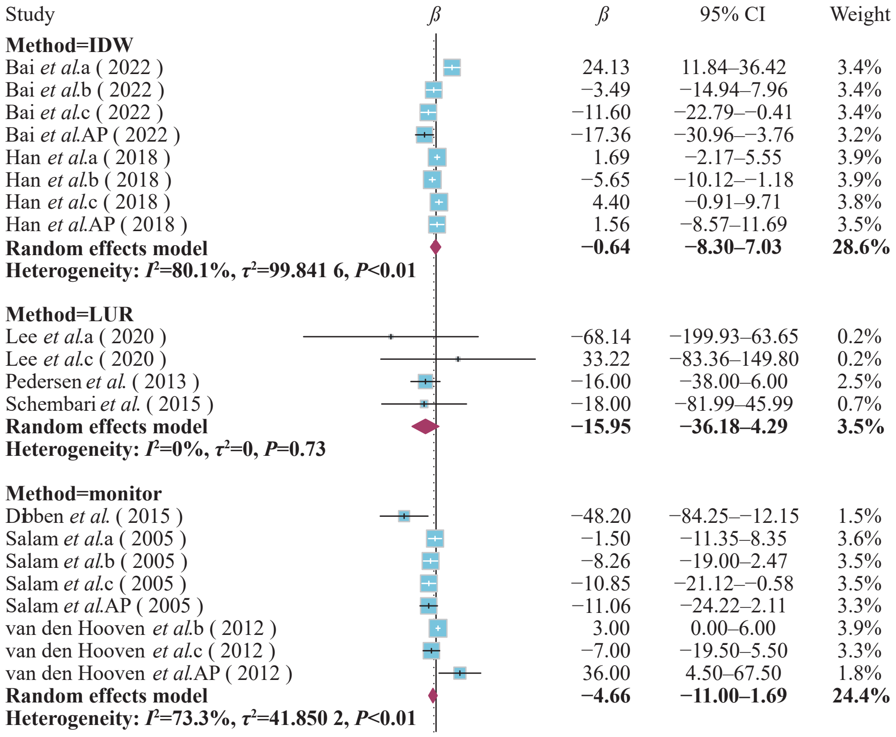

(Continued)

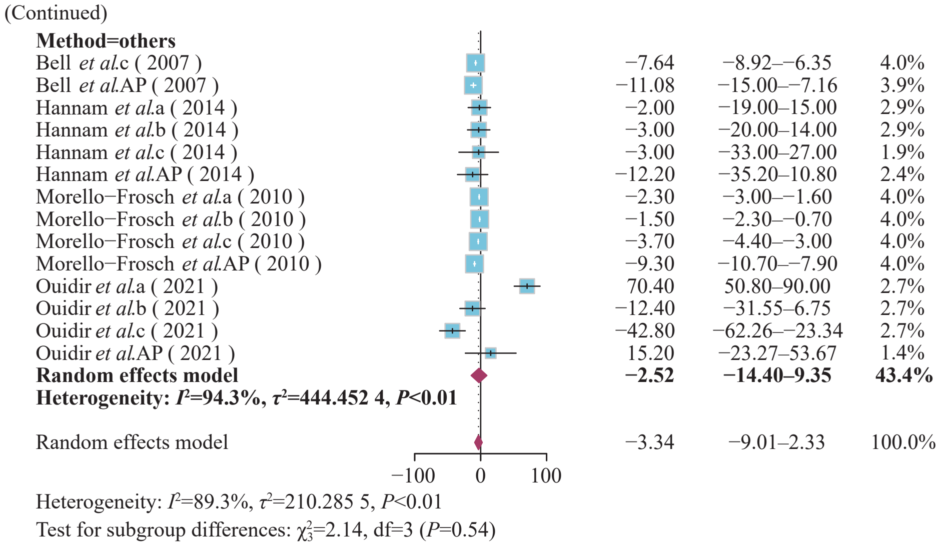

Supplementary Fig. 8 **Forest plot of pooled estimates of changes (regression coefficients [*β*] with 95% confidence interval [CI]) in birth weight associated with PM_{10} exposure during the entire pregnancy, stratified by exposure assessment methods subgroup, analyzed using the random effects model with the subgroup specified as exposure assessment methods.** a: first trimester, b: second trimester, c: third trimester, AP: entire pregnancy.

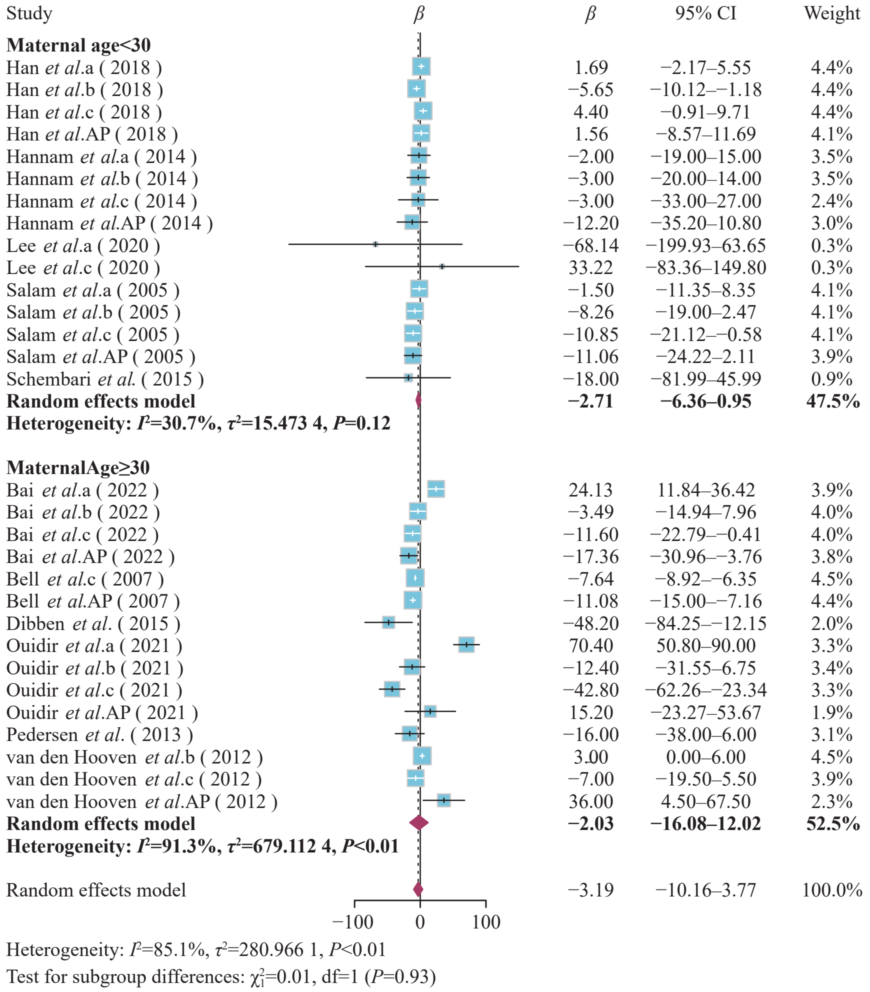

Supplementary Fig. 9 **Forest plot of pooled estimates of changes (regression coefficients [*β*] with 95% confidence interval [CI]) in birth weight associated with PM_{10} exposure during the entire pregnancy, stratified by maternal age subgroup, analyzed using the random effects model with the subgroup specified as maternal age.** a: first trimester, b: second trimester, c: third trimester, AP: entire pregnancy.

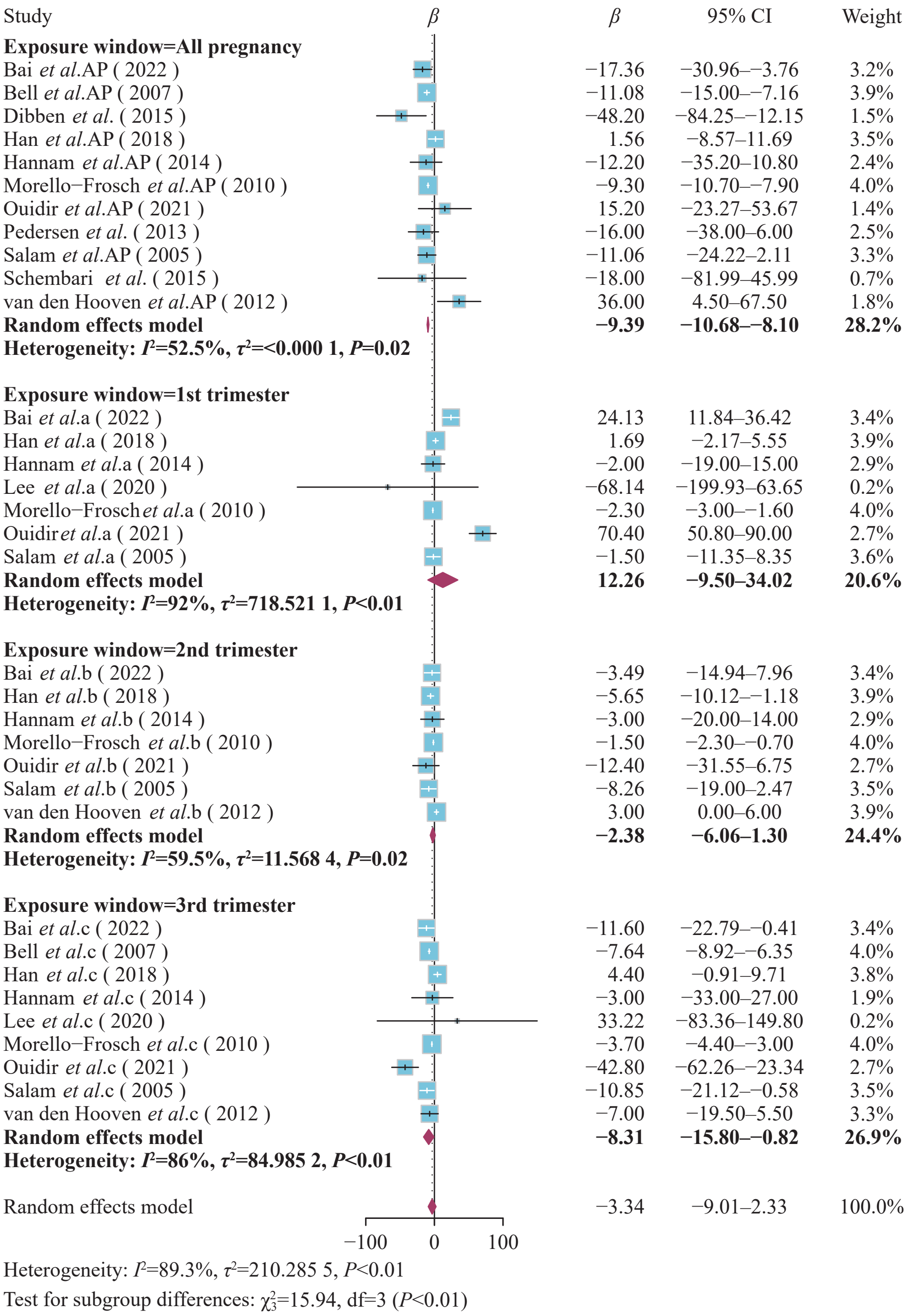

Supplementary Fig. 10 **Forest plot of pooled estimates of changes (regression coefficients [*β*] with 95% confidence interval [CI]) in birth weight associated with PM_{10} exposure during the entire pregnancy, stratified by pregnancy exposure window subgroup, analyzed using the random effects model with the subgroup specified as pregnancy exposure window.** a: first trimester, b: second trimester, c: third trimester, AP: entire pregnancy.

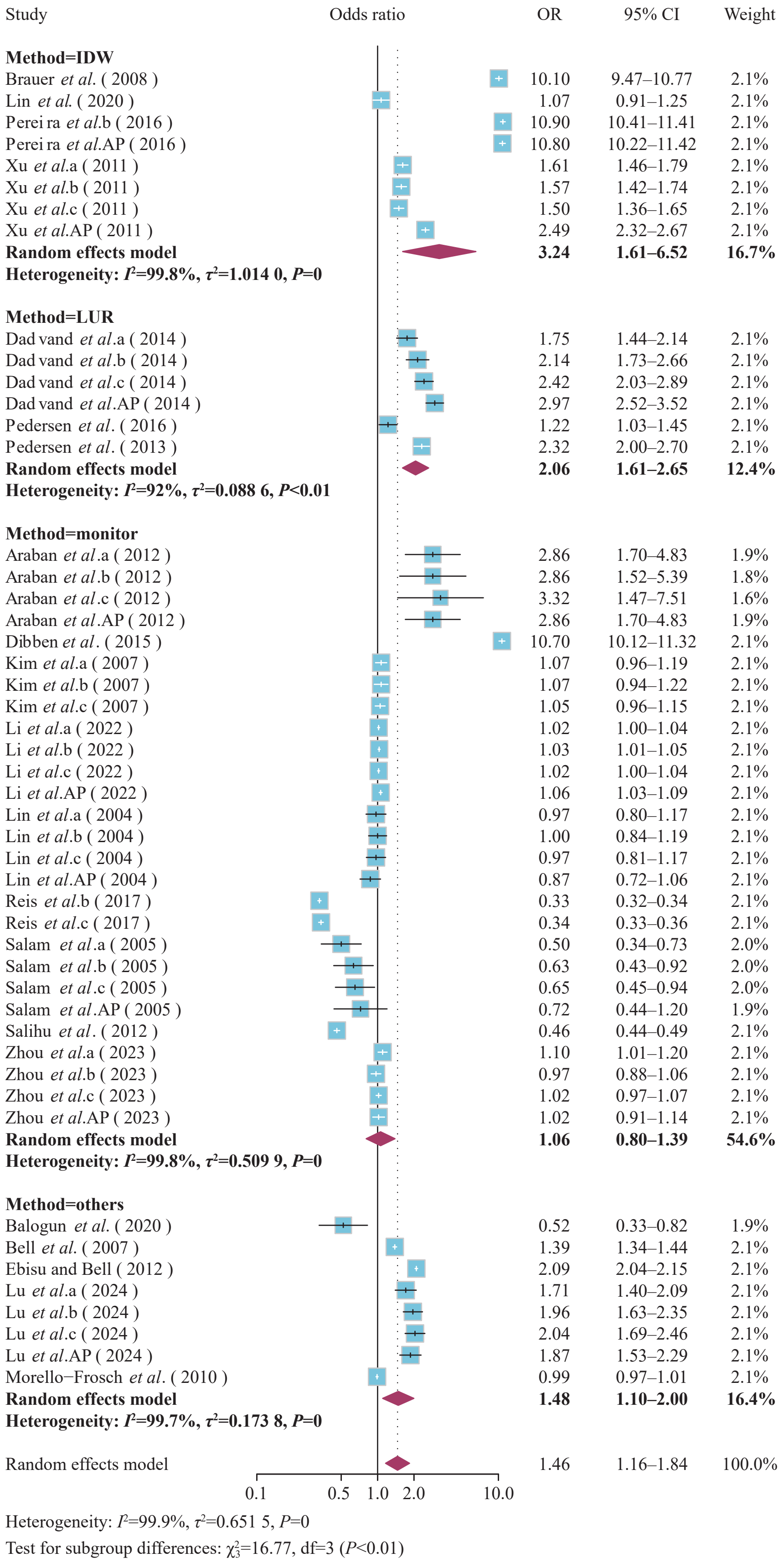

Supplementary Fig. 11 **Forest plot of pooled associations between PM_{10} exposure during the entire pregnancy and low birth weight risk (odds ratio [OR] with 95% confidence interval [CI]) in exposure assessment methods subgroups, analyzed using the random effects model with the subgroup specified as exposure assessment methods.** a: first trimester, b: second trimester, c: third trimester, AP: entire pregnancy.

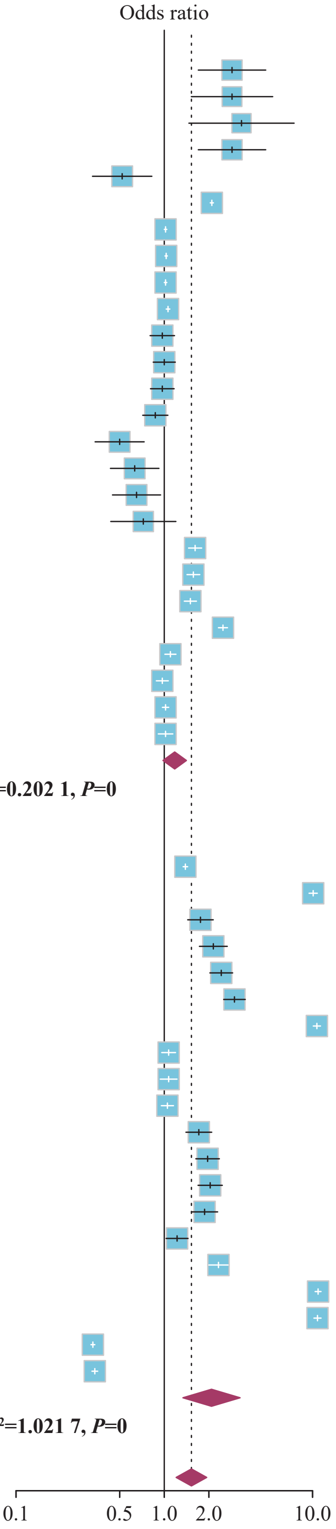

Study	OR	95% CI	Weight
**Maternal age<30**			
Araban *et al*.a (2012)	2.86	1.70–4.83	2.0%
Araban *et al*.b (2012)	2.86	1.52–5.39	1.9%
Araban *et al*.c (2012)	3.32	1.47–7.51	1.8%
Araban *et al*.AP (2012)	2.86	1.70–4.83	2.0%
Balogun *et al*. (2020)	0.52	0.33–0.82	2.1%
Ebisu and Bell (2012)	2.09	2.04–2.15	2.2%
Li *et al*.a (2022)	1.02	1.00–1.04	2.2%
Li *et al*.b (2022)	1.03	1.01–1.05	2.2%
Li *et al*.c (2022)	1.02	1.00–1.04	2.2%
Li *et al*.AP (2022)	1.06	1.03–1.09	2.2%
Lin *et al*.a (2004)	0.97	0.80–1.17	2.2%
Lin *et al*.b (2004)	1.00	0.84–1.19	2.2%
Lin *et al*.c (2004)	0.97	0.81–1.17	2.2%
Lin *et al*.AP (2004)	0.87	0.72–1.06	2.2%
Salam *et al*.a (2005)	0.50	0.34–0.73	2.1%
Salam *et al*.b (2005)	0.63	0.43–0.92	2.1%
Salam *et al*.c (2005)	0.65	0.45–0.94	2.1%
Salam *et al*.AP (2005)	0.72	0.44–1.20	2.0%
Xu *et al*.a (2011)	1.61	1.46–1.79	2.2%
Xu *et al*.b (2011)	1.57	1.42–1.74	2.2%
Xu *et al*.c (2011)	1.50	1.36–1.65	2.2%
Xu *et al*.AP (2011)	2.49	2.32–2.67	2.2%
Zhou *et al*.a (2023)	1.10	1.01–1.20	2.2%
Zhou *et al*.b (2023)	0.97	0.88–1.06	2.2%
Zhou *et al*.c (2023)	1.02	0.97–1.07	2.2%
Zhou *et al*.AP (2023)	1.02	0.91–1.14	2.2%
**Random effects model**	**1.18**	**0.98–1.41**	**55.8%**
**Heterogeneity: I^2=99.3%, τ^2=0.202 1, *P*=0**			
**MaternalAge≥30**			
Bell *et al.* (2007)	1.39	1.34–1.44	2.2%
Brauer *et al.* (2008)	10.10	9.47–10.77	2.2%
Dadvand *et al*.a (2014)	1.75	1.44–2.14	2.2%
Dadvand *et al*.b (2014)	2.14	1.73–2.66	2.2%
Dadvand *et al*.c (2014)	2.42	2.03–2.89	2.2%
Dadvand *et al*.AP (2014)	2.97	2.52–3.52	2.2%
Dibben *et al.* (2015)	10.70	10.12–11.32	2.2%
Kim *et al*.a (2007)	1.07	0.96–1.19	2.2%
Kim *et al*.b (2007)	1.07	0.94–1.22	2.2%
Kim *et al*.c (2007)	1.05	0.96–1.15	2.2%
Lu *et al*.a (2024)	1.71	1.40–2.09	2.2%
Lu *et al*.b (2024)	1.96	1.63–2.35	2.2%
Lu *et al*.c (2024)	2.04	1.69–2.46	2.2%
Lu et al.AP (2024)	1.87	1.53–2.29	2.2%
Pedersen *et al.* (2016)	1.22	1.03–1.45	2.2%
Pedersen *et al.* (2013)	2.32	2.00–2.70	2.2%
Pereira *et al*.b (2016)	10.90	10.41–11.41	2.2%
Pereira *et al*.AP (2016)	10.80	10.22–11.42	2.2%
Reis *et al*.b (2017)	0.33	0.32–0.34	2.2%
Reis *et al*.c (2017)	0.34	0.33–0.36	2.2%
**Random effects model**	**2.08**	**1.34–3.25**	**44.2%**
**Heterogeneity: I^2 = 99.9%, τ^2=1.021 7, *P*=0**			
Random effects model	1.52	1.20–1.93	100.0%

Heterogeneity: I^2=99.9%, τ^2=0.658 2, *P*=0

Test for subgroup differences: χ^2_1=5.45, df=1 (*P*=0.02)

Supplementary Fig. 12 **Forest plot of pooled associations between PM_{10} exposure during the entire pregnancy and low birth weight risk (odds ratio [OR] with 95% confidence interval [CI]) in maternal age subgroups, analyzed using the random effects model with the subgroup specified as maternal age.** a: first trimester, b: second trimester, c: third trimester, AP: entire pregnancy.

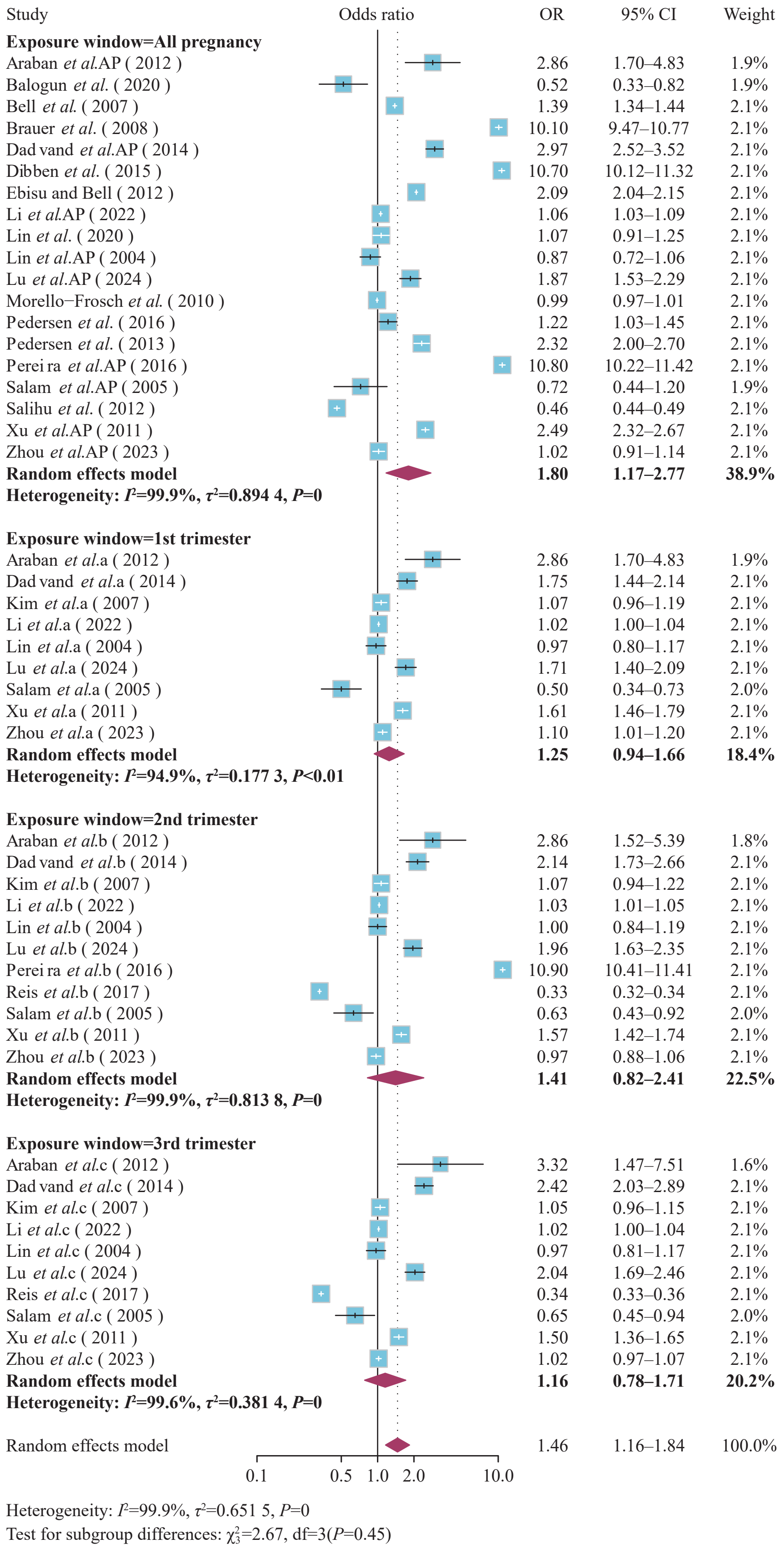

Supplementary Fig. 13 **Forest plot of pooled associations between PM_{10} exposure during the entire pregnancy and low birth weight risk (odds ratio [OR] with 95% confidence interval [CI]) in pregnancy exposure window subgroups, analyzed using the random effects model with the subgroup specified as pregnancy exposure window.** a: first trimester, b: second trimester, c: third trimester, AP: entire pregnancy.

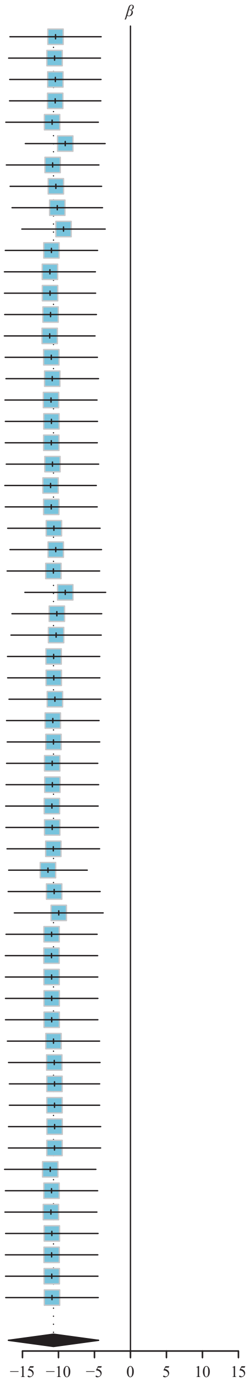

Study	β	95% CI	P−value	Tau2	Tau	I^2
Omitting Bai *et al*.a (2022)	−10.41	−16.75–−4.07	<0.01	537.856 1	23.191 7	99%
Omitting Bai *et al*.b (2022)	−10.54	−16.93–−4.16	<0.01	544.764 8	23.340 2	99%
Omitting Bai *et al*.c (2022)	−10.43	−16.78–−4.08	<0.01	538.880 0	23.213 8	99%
Omitting Bai *et al*.AP (2022)	−10.46	−16.80–−4.11	<0.01	539.406 6	23.225 1	99%
Omitting Balak rishnan *et al.* (2018)	−10.89	−17.32–−4.46	<0.01	551.706 3	23.488 4	99%
Omitting Balidemaj *et al.* (2022)	−9.06	−14.61–−3.51	<0.01	407.147 7	20.177 9	99%
Omitting Basu *et al*. (2014)	−10.81	−17.25–−4.38	<0.01	552.530 7	23.506 0	99%
Omitting Bell *et al*.b (2007)	−10.36	−16.72–−4.00	<0.01	538.608 9	23.207 9	99%
Omitting Bell *et al*.c (2007)	−10.17	−16.45–−3.88	<0.01	525.514 6	22.924 1	99%
Omitting Bell *et al*.AP (2007)	−9.30	−15.09–−3.50	<0.01	440.448 3	20.986 9	99%
Omitting Dong *et al* . (2020)	−10.98	−17.40–−4.56	<0.01	548.965 9	23.430 0	99%
Omitting Du *et al*.a (2022)	−11.19	−17.51–−4.86	<0.01	532.215 2	23.069 8	99%
Omitting Du *et al*.b (2022)	−11.17	−17.51–−4.84	<0.01	534.196 4	23.112 7	99%
Omitting Du *et al*.c (2022)	−11.10	−17.48–−4.72	<0.01	541.408 5	23.268 2	99%
Omitting Du *et al*.AP (2022)	−11.22	−17.52–−4.91	<0.01	528.210 4	22.982 8	98%
Omitting Han *et al*.a (2018)	−11.00	−17.41–−4.58	<0.01	548.062 4	23.410 7	99%
Omitting Han *et al*.b (2018)	−10.87	−17.30–−4.43	<0.01	551.902 5	23.492 6	99%
Omitting Han *et al*.c (2018)	−11.04	−17.44–−4.64	<0.01	545.721 7	23.360 7	99%
Omitting Han *et al*.AP (2018)	−10.98	−17.39–−4.57	<0.01	547.785 4	23.404 8	99%
Omitting Hannam *et al*.a (2014)	−11.00	−17.39–−4.60	<0.01	546.196 7	23.370 9	99%
Omitting Hannam *et al*.b (2014)	−10.84	−17.26–−4.41	<0.01	550.825 5	23.469 7	99%
Omitting Hannam *et al*.c (2014)	−11.10	−17.46–−4.74	<0.01	539.404 5	23.225 1	99%
Omitting Hannam *et al*.AP (2014)	−11.00	−17.39–−4.61	<0.01	545.264 8	23.350 9	99%
Omitting Hyder *et al*.a (2014)	−10.64	−17.06–−4.21	<0.01	550.346 7	23.459 5	99%
Omitting Hyder *et al*.b (2014)	−10.38	−16.74–−4.02	<0.01	539.313 5	23.223 1	99%
Omitting Hyder *et al*.c (2014)	−10.71	−17.13–−4.28	<0.01	551.554 1	23.485 2	99%
Omitting Hyder *et al*.AP (2014)	−9.06	−14.67–−3.44	<0.01	412.012 4	20.298 1	99%
Omitting Johnson *et al*.b (2022)	−10.23	−16.46–−3.99	<0.01	522.304 1	22.854 0	99%
Omitting Johnson *et al*.c (2022)	−10.33	−16.60–−4.06	<0.01	528.782 0	22.995 3	99%
Omitting Keller *et al*.a (2017)	−10.67	−17.09–−4.26	<0.01	549.464 8	23.440 7	99%
Omitting Keller *et al*.b (2017)	−10.65	−17.06–−4.24	<0.01	548.972 1	23.430 2	99%
Omitting Keller *et al*.c (2017)	−10.50	−16.88–−4.11	<0.01	543.927 6	23.322 3	99%
Omitting Kloog *et al*.c (2012)	−10.79	−17.22–−4.35	<0.01	552.355 0	23.502 2	99%
Omitting Kloog *et al*.AP (2012)	−10.69	−17.12–−4.27	<0.01	551.330 9	23.480 4	99%
Omitting Melody *et al.* (2019)	−10.87	−17.21–−4.53	<0.01	541.089 2	23.261 3	99%
Omitting Morello−Frosch *et al*.a (2010)	−10.85	−17.28–−4.41	<0.01	552.344 9	23.502 0	99%
Omitting Morello−Frosch *et al*.b (2010)	−10.91	−17.34–−4.48	<0.01	551.220 9	23.478 1	99%
Omitting Morello−Frosch *et al*.c (2010)	−10.87	−17.31–−4.44	<0.01	552.013 7	23.495 0	99%
Omitting Morello−Frosch *et al*.AP (2010)	−10.72	−17.15–−4.28	<0.01	552.095 3	23.496 7	99%
Omitting Ouidir *et al*.a (2021)	−11.46	−16.93–−6.00	<0.01	388.554 6	19.711 8	99%
Omitting Ouidir *et al*.b (2021)	−10.60	−16.99–−4.21	<0.01	546.581 8	23.379 1	99%
Omitting Ouidir *et al*.c (2021)	−9.96	−16.14–−3.79	<0.01	506.778 7	22.511 7	99%
Omitting Ouidir *et al*.AP (2021)	−10.96	−17.29–−4.62	<0.01	539.039 7	23.217 2	99%
Omitting Payne−Sturges *et al*.a (2022)	−10.97	−17.39–−4.55	<0.01	549.198 6	23.435 0	99%
Omitting Payne−Sturges *et al*.b (2022)	−10.95	−17.38–−4.53	<0.01	549.760 4	23.447 0	99%
Omitting Payne−Sturges *et al*.c (2022)	−10.94	−17.37–−4.52	<0.01	550.178 2	23.455 9	99%
Omitting Payne−Sturges *et al*.AP (2022)	−10.94	−17.37–−4.52	<0.01	550.154 4	23.455 4	99%
Omitting Pedersen *et al*. (2013)	−10.69	−17.10–−4.28	<0.01	549.116 0	23.433 2	99%
Omitting Schembari *et al*. (2015)	−10.58	−16.95–−4.22	<0.01	542.966 3	23.301 6	99%
Omitting Soesanti *et al*.a (2023)	−10.55	−16.83–−4.27	<0.01	533.025 7	23.087 3	99%
Omitting Soesanti *et al*.b (2023)	−10.55	−16.83–−4.27	<0.01	533.025 7	23.087 3	99%
Omitting Stieb *et al.* (2016)	−10.54	−16.94–−4.13	<0.01	547.172 2	23.391 7	99%
Omitting Stieb *et al.* (2016)	−10.55	−16.96–−4.14	<0.01	547.723 3	23.403 5	99%
Omitting Vini koor−Imler *et al*.a (2014)	−11.14	−17.49–−4.80	<0.01	536.478 2	23.162 0	99%
Omitting Vini koor−Imler *et al*.b (2014)	−10.97	−17.38–−4.55	<0.01	548.869 7	23.428 0	99%
Omitting Vini koor−Imler *et al*.c (2014)	−11.05	−17.44–−4.66	<0.01	544.541 6	23.335 4	99%
Omitting Wu *et al*.a (2021)	−10.92	−17.35–−4.49	<0.01	550.946 4	23.472 2	99%
Omitting Wu *et al*.b (2021)	−10.95	−17.38–−4.53	<0.01	549.919 6	23.450 4	99%
Omitting Wu *et al*.c (2021)	−10.93	−17.36–−4.51	<0.01	550.583 0	23.464 5	99%
Omitting Wu *et al*.AP (2021)	−10.90	−17.33–−4.47	<0.01	551.465 2	23.483 3	99%
Random effects model	−10.69	−16.97–−4.42	<0.01	533.667 2	23.101 2	99%

Supplementary Fig. 14 **Sensitivity analysis of pooled estimates of changes (regression coefficients [β] with 95% confidence interval [CI]) in birth weight associated with $PM_{2.5}$ exposure during the entire pregnancy, analyzed using the random effects model.** a: first trimester, b: second trimester, c: third trimester, AP: entire pregnancy.

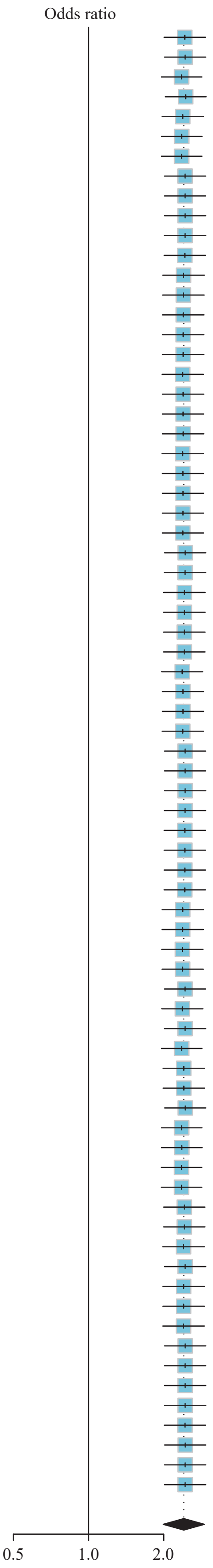

Study	OR	95% CI	P−value	Tau2	Tau	I^2
Omitting Ahmad *et al.* (2022)	2.43	2.01–2.94	<0.01	0.682 5	0.826 1	100%
Omitting Balakrishnan *et al.* (2018)	2.44	2.01–2.95	<0.01	0.678 0	0.823 4	100%
Omitting Balidemaj *et al.* (2022)	2.36	1.96–2.85	<0.01	0.656 9	0.810 5	100%
Omitting Balogun *et al.* (2020)	2.45	2.03–2.96	<0.01	0.665 8	0.816 0	100%
Omitting Bell *et al.* (2007)	2.39	1.97–2.89	<0.01	0.682 0	0.825 8	100%
Omitting Brauer *et al.*IDW (2008)	2.36	1.96–2.85	<0.01	0.660 7	0.812 8	100%
Omitting Brauer *et al.*LUR (2008)	2.36	1.96–2.85	<0.01	0.658 6	0.811 5	100%
Omitting Chen *et al.*a (2020)	2.44	2.01–2.95	<0.01	0.678 7	0.823 8	100%
Omitting Chen *et al.*b (2020)	2.44	2.01–2.95	<0.01	0.678 5	0.823 7	100%
Omitting Chen *et al.*c (2020)	2.44	2.01–2.95	<0.01	0.678 2	0.823 6	100%
Omitting Chen *et al.*AP (2020)	2.44	2.01–2.95	<0.01	0.678 7	0.823 8	100%
Omitting Coker *et al.* (2015)	2.43	2.01–2.94	<0.01	0.681 5	0.825 5	100%
Omitting Dadvand *et al.*a (2014)	2.40	1.98–2.91	<0.01	0.688 1	0.829 5	100%
Omitting Dadvand *et al.*b (2014)	2.40	1.98–2.90	<0.01	0.687 4	0.829 1	100%
Omitting Dadvand *et al.*c (2014)	2.40	1.98–2.90	<0.01	0.686 8	0.828 8	100%
Omitting Dadvand *et al.*AP (2014)	2.39	1.98–2.90	<0.01	0.685 8	0.828 1	100%
Omitting Ebisu and Bell (2012)	2.39	1.98–2.90	<0.01	0.685 9	0.828 2	100%
Omitting Gray *et al.* (2014)	2.38	1.97–2.88	<0.01	0.680 7	0.825 1	100%
Omitting Ha *et al.*a (2014)	2.39	1.98–2.90	<0.01	0.685 5	0.828 0	100%
Omitting Ha *et al.*b (2014)	2.39	1.97–2.90	<0.01	0.685 1	0.827 7	100%
Omitting Ha *et al.*c (2014)	2.39	1.98–2.90	<0.01	0.686 1	0.828 3	100%
Omitting Ha *et al.*AP (2014)	2.38	1.97–2.88	<0.01	0.680 8	0.825 1	100%
Omitting Hyder *et al.*a (2014)	2.39	1.97–2.89	<0.01	0.683 7	0.826 9	100%
Omitting Hyder *et al.*b (2014)	2.39	1.97–2.89	<0.01	0.684 5	0.827 4	100%
Omitting Hyder *et al.*c (2014)	2.39	1.97–2.89	<0.01	0.684 2	0.827 2	100%
Omitting Hyder *et al.*AP (2014)	2.39	1.97–2.89	<0.01	0.683 1	0.826 5	100%
Omitting Kloog *et al.*c (2012)	2.44	2.02–2.95	<0.01	0.677 3	0.823 0	100%
Omitting Kloog *et al.*AP (2012)	2.44	2.01–2.95	<0.01	0.678 9	0.824 0	100%
Omitting Laurent *et al.*a (2014)	2.42	2.00–2.93	<0.01	0.687 0	0.828 9	100%
Omitting Laurent *et al.*b (2014)	2.42	2.00–2.93	<0.01	0.686 9	0.828 8	100%
Omitting Laurent *et al.*c (2014)	2.42	2.00–2.93	<0.01	0.687 2	0.829 0	100%
Omitting Laurent *et al.*AP (2014)	2.42	2.00–2.93	<0.01	0.687 3	0.829 0	100%
Omitting Laurent *et al.* (2013)	2.37	1.96–2.87	<0.01	0.671 3	0.819 3	100%
Omitting Lavigne *et al.*b (2016)	2.39	1.97–2.89	<0.01	0.684 2	0.827 2	100%
Omitting Lavigne *et al.*c (2016)	2.39	1.97–2.89	<0.01	0.684 5	0.827 3	100%
Omitting Lavigne *et al.*AP (2016)	2.39	1.97–2.89	<0.01	0.682 1	0.825 9	100%
Omitting Li *et al.*a (2022)	2.44	2.01–2.95	<0.01	0.678 2	0.823 6	100%
Omitting Li *et al.*b (2022)	2.44	2.01–2.95	<0.01	0.678 2	0.823 6	100%
Omitting Li *et al.*c (2022)	2.44	2.01–2.95	<0.01	0.678 2	0.823 6	100%
Omitting Li *et al.*AP (2022)	2.44	2.01–2.95	<0.01	0.679 2	0.824 1	100%
Omitting Liang *et al.*a (2019)	2.43	2.01–2.94	<0.01	0.681 6	0.825 6	100%
Omitting Liang *et al.*b (2019)	2.43	2.01–2.94	<0.01	0.681 3	0.825 4	100%
Omitting Liang *et al.*c (2019)	2.43	2.01–2.94	<0.01	0.681 6	0.825 6	100%
Omitting Lin *et al.* (2020)	2.43	2.01–2.94	<0.01	0.682 3	0.826 0	100%
Omitting Lu *et al.*a (2024)	2.38	1.97–2.89	<0.01	0.681 6	0.825 6	100%
Omitting Lu *et al.*b (2024)	2.38	1.97–2.88	<0.01	0.680 4	0.824 9	100%
Omitting Lu *et al.*c (2024)	2.38	1.97–2.87	<0.01	0.676 3	0.822 4	100%
Omitting Lu *et al.*AP (2024)	2.38	1.97–2.88	<0.01	0.679 8	0.824 5	100%
Omitting Melody *et al.* (2019)	2.44	2.01–2.95	<0.01	0.678 2	0.823 5	100%
Omitting Meng *et al.* (2023)	2.38	1.96–2.87	<0.01	0.675 5	0.821 9	100%
Omitting Morello−Frosch *et al.* (2010)	2.44	2.01–2.95	<0.01	0.678 5	0.823 7	100%
Omitting Ottone *et al.* (2020)	2.36	1.96–2.85	<0.01	0.659 5	0.812 1	100%
Omitting Pedersen *et al.* (2016)	2.41	1.99–2.92	<0.01	0.688 7	0.829 9	100%
Omitting Pedersen *et al.* (2013)	2.41	1.99–2.92	<0.01	0.688 7	0.829 9	100%
Omitting Salihu *et al.* (2012)	2.44	2.02–2.95	<0.01	0.676 2	0.822 3	100%
Omitting Slama *et al.*a (2007)	2.36	1.96–2.85	<0.01	0.658 9	0.811 7	100%
Omitting Slama *et al.*b (2007)	2.36	1.96–2.85	<0.01	0.662 5	0.813 9	100%
Omitting Slama *et al.*c (2007)	2.36	1.96–2.84	<0.01	0.654 6	0.809 1	100%
Omitting Slama *et al.*AP (2007)	2.36	1.96–2.84	<0.01	0.655 0	0.809 3	100%
Omitting Soesanti *et al.*a (2023)	2.42	2.00–2.93	<0.01	0.683 8	0.826 9	100%
Omitting Soesanti *et al.*b (2023)	2.42	2.00–2.93	<0.01	0.683 8	0.826 9	100%
Omitting Stieb *et al.* (2016)	2.40	1.98–2.91	<0.01	0.688 3	0.829 6	100%
Omitting Stieb *et al.* (2016)	2.44	2.01–2.95	<0.01	0.677 8	0.823 3	100%
Omitting Vinikoor−Imler *et al.*a (2014)	2.40	1.98–2.91	<0.01	0.688 5	0.829 8	100%
Omitting Vinikoor−Imler *et al.*b (2014)	2.40	1.98–2.91	<0.01	0.688 5	0.829 8	100%
Omitting Vinikoor−Imler *et al.*c (2014)	2.40	1.98–2.91	<0.01	0.688 3	0.829 6	100%
Omitting Zhang *et al.*a (2022)	2.44	2.02–2.95	<0.01	0.677 2	0.822 9	100%
Omitting Zhang *et al.*b (2022)	2.44	2.01–2.95	<0.01	0.678 2	0.823 6	100%
Omitting Zhang *et al.*c (2022)	2.44	2.02–2.95	<0.01	0.677 7	0.823 3	100%
Omitting Zhang *et al.*AP (2022)	2.44	2.01–2.95	<0.01	0.678 0	0.823 4	100%
Omitting Zhou *et al.*a (2023)	2.43	2.01–2.95	<0.01	0.679 8	0.824 5	100%
Omitting Zhou *et al.*b (2023)	2.44	2.02–2.95	<0.01	0.676 8	0.822 7	100%
Omitting Zhou *et al.*c (2023)	2.44	2.01–2.95	<0.01	0.678 0	0.823 4	100%
Omitting Zhou *et al.*AP (2023)	2.44	2.01–2.95	<0.01	0.678 1	0.823 4	100%
Random effects model	2.41	1.99–2.91	<0.01	0.679 1	0.824 1	100%

Supplementary Fig. 15 **Sensitivity analysis of pooled associations between $PM_{2.5}$ exposure during the entire pregnancy and low birth weight risk (odds ratio [OR] with 95% confidence interval [CI]), analyzed using the random effects model.** a: first trimester, b: second trimester, c: third trimester, AP: entire pregnancy.

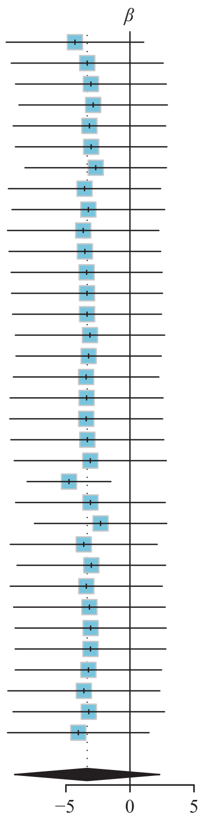

Study	β	95% CI	P−value	Tau2	Tau	I^2
Omitting Bai *et al.*a (2022)	−4.29	−9.66–1.08	0.12	177.378 4	13.318 3	89%
Omitting Bai *et al.*b (2022)	−3.34	−9.27–2.60	0.27	224.840 2	14.994 7	90%
Omitting Bai *et al.*c (2022)	−3.05	−8.93–2.84	0.31	220.724 0	14.856 8	90%
Omitting Bai *et al.*AP (2022)	−2.87	−8.67–2.92	0.33	213.296 1	14.604 7	89%
Omitting Bell *et al.*c (2007)	−3.17	−9.12–2.79	0.30	225.509 1	15.017 0	88%
Omitting Bell *et al.*AP (2007)	−3.03	−8.94–2.89	0.32	222.037 6	14.900 9	89%
Omitting Dbben *et al.* (2015)	−2.67	−8.20–2.85	0.34	194.389 1	13.942 3	89%
Omitting Han *et al.*a (2018)	−3.54	−9.49–2.40	0.24	224.782 7	14.992 8	89%
Omitting Han *et al.*b (2018)	−3.25	−9.21–2.71	0.29	226.240 9	15.041 3	90%
Omitting Han *et al.*c (2018)	−3.65	−9.56–2.26	0.23	221.959 9	14.898 3	89%
Omitting Han *et al.*AP (2018)	−3.52	−9.44–2.40	0.24	223.731 7	14.957 7	90%
Omitting Hannam *et al.*a (2014)	−3.38	−9.28–2.51	0.26	222.756 5	14.925 0	90%
Omitting Hannam *et al.*b (2014)	−3.35	−9.25–2.54	0.27	222.830 7	14.927 5	90%
Omitting Hannam *et al.*c (2014)	−3.35	−9.16–2.47	0.26	218.404 7	14.778 5	90%
Omitting Hannam *et al.*AP (2014)	−3.12	−8.95–2.71	0.29	218.268 9	14.773 9	90%
Omitting Lee *et al.*a (2020)	−3.23	−8.90–2.45	0.27	210.408 4	14.505 5	90%
Omitting Lee *et al.*c (2020)	−3.42	−9.10–2.26	0.24	210.917 7	14.523 0	90%
Omitting Morello−Frosch *et al.*a (2010)	−3.38	−9.36–2.59	0.27	226.929 2	15.064 2	89%
Omitting Morello−Frosch *et al.*b (2010)	−3.42	−9.39–2.55	0.26	226.743 1	15.058 0	89%
Omitting Morello−Frosch *et al.*c (2010)	−3.33	−9.30–2.65	0.27	227.003 5	15.066 6	90%
Omitting Morello−Frosch *et al.*AP (2010)	−3.10	−9.04–2.84	0.31	224.125 3	14.970 8	86%
Omitting Ouidir *et al.*a (2021)	−4.76	−8.03–−1.48	<0.01	50.947 5	7.137 8	87%
Omitting Ouidir *et al.*b (2021)	−3.09	−8.93–2.76	0.30	218.823 0	14.792 7	90%
Omitting Ouidir *et al.*c (2021)	−2.30	−7.46–2.87	0.38	163.038 8	12.768 7	89%
Omitting Ouidir *et al.*AP (2021)	−3.61	−9.35–2.13	0.22	212.909 9	14.591 4	90%
Omitting Pedersen *et al.* (2013)	−3.02	−8.82–2.79	0.31	215.824 8	14.691 0	90%
Omitting Salam *et al.*a (2005)	−3.41	−9.35–2.53	0.26	225.137 7	15.004 6	90%
Omitting Salam *et al.*b (2005)	−3.16	−9.08–2.76	0.29	223.555 1	14.951 8	90%
Omitting Salam *et al.*c (2005)	−3.07	−8.97–2.83	0.31	221.605 1	14.886 4	90%
Omitting Salam *et al.*AP (2005)	−3.08	−8.96–2.80	0.30	220.925 4	14.863 6	90%
Omitting Schembari *et al.* (2015)	−3.24	−8.96–2.47	0.27	212.639 2	14.582 2	90%
Omitting van den Hooven *et al.*b (2012)	−3.60	−9.53–2.34	0.23	223.681 7	14.956 0	89%
Omitting van den Hooven *et al.*c (2012)	−3.22	−9.13–2.70	0.29	223.707 2	14.956 8	90%
Omitting van den Hooven *et al.*AP (2012)	−4.03	−9.56–1.49	0.15	193.863 3	13.923 5	89%
Random effects model	−3.34	−9.01–2.33	0.25	210.285 5	14.501 2	89%

Supplementary Fig. 16 **Sensitivity analysis of pooled estimates of changes (regression coefficients [*β*] with 95% confidence interval [CI]) in birth weight associated with PM_{10} exposure during the entire pregnancy, analyzed using the random effects model.** a: first trimester, b: second trimester, c: third trimester, AP: entire pregnancy.

Study	Odds ratio	OR	95% CI	*P*−value	Tau2	Tau	I^2
Omitting Araban *et al*.a (2012)		1.44	1.15–1.82	<0.01	0.656 2	0.810 0	100%
Omitting Araban *et al*.b (2012)		1.44	1.15–1.82	<0.01	0.656 3	0.810 1	100%
Omitting Araban *et al*.c (2012)		1.44	1.15–1.82	<0.01	0.653 5	0.808 4	100%
Omitting Araban *et al*.AP (2012)		1.44	1.15–1.82	<0.01	0.656 2	0.810 0	100%
Omitting Balogun *et al.* (2020)		1.49	1.19–1.88	<0.01	0.644 4	0.802 8	100%
Omitting Bell *et al.* (2007)		1.46	1.16–1.85	<0.01	0.665 7	0.815 9	100%
Omitting Brauer *et al.* (2008)		1.40	1.13–1.75	<0.01	0.581 6	0.762 7	100%
Omitting Dad vand *et al*.a (2014)		1.46	1.15–1.84	<0.01	0.664 9	0.815 4	100%
Omitting Dad vand *et al*.b (2014)		1.45	1.15–1.83	<0.01	0.662 4	0.813 9	100%
Omitting Dad vand *et al*.c (2014)		1.45	1.15–1.83	<0.01	0.660 0	0.812 4	100%
Omitting Dad vand *et al*.AP (2014)		1.44	1.14–1.82	<0.01	0.654 5	0.809 0	100%
Omitting Dibben *et al.* (2015)		1.40	1.13–1.74	<0.01	0.576 4	0.759 2	100%
Omitting Ebisu and Bell (2012)		1.45	1.15–1.83	<0.01	0.662 9	0.814 2	100%
Omitting Kim *et al*.a (2007)		1.47	1.17–1.86	<0.01	0.663 6	0.814 6	100%
Omitting Kim *et al*.b (2007)		1.47	1.17–1.86	<0.01	0.663 6	0.814 6	100%
Omitting Kim *et al*.c (2007)		1.47	1.17–1.86	<0.01	0.663 3	0.814 5	100%
Omitting Li *et al*.a (2022)		1.47	1.17–1.86	<0.01	0.662 9	0.814 2	100%
Omitting Li *et al*.b (2022)		1.47	1.17–1.86	<0.01	0.663 1	0.814 3	100%
Omitting Li *et al*.c (2022)		1.47	1.17–1.86	<0.01	0.662 9	0.814 2	100%
Omitting Li *et al*.AP (2022)		1.47	1.17–1.86	<0.01	0.663 5	0.814 6	100%
Omitting Lin *et al.* (2020)		1.47	1.17–1.86	<0.01	0.663 5	0.814 6	100%
Omitting Lin *et al*.a (2004)		1.48	1.17–1.86	<0.01	0.662 0	0.813 6	100%
Omitting Lin *et al*.b (2004)		1.47	1.17–1.86	<0.01	0.662 5	0.813 9	100%
Omitting Lin *et al*.c (2004)		1.48	1.17–1.86	<0.01	0.662 0	0.813 6	100%
Omitting Lin *et al*.AP (2004)		1.48	1.17–1.87	<0.01	0.659 7	0.8122	100%
Omitting Lu *et al*.a (2024)		1.46	1.15–1.84	<0.01	0.665 0	0.815 5	100%
Omitting Lu *et al*.b (2024)		1.45	1.15–1.84	<0.01	0.663 7	0.814 7	100%
Omitting Lu *et al*.c (2024)		1.45	1.15–1.83	<0.01	0.663 2	0.814 4	100%
Omitting Lu *et al*.AP (2024)		1.46	1.15–1.84	<0.01	0.664 3	0.815 0	100%
Omitting Morello−Frosch *et al.* (2010)		1.48	1.17–1.86	<0.01	0.662 4	0.813 9	100%
Omitting Pedersen *et al.* (2016)		1.47	1.16–1.85	<0.01	0.664 9	0.815 4	100%
Omitting Pedersen *et al.* (2013)		1.45	1.15–1.83	<0.01	0.661 0	0.813 0	100%
Omitting Perei ra *et al*.b (2016)		1.40	1.13–1.74	<0.01	0.574 7	0.758 1	100%
Omitting Perei ra *et al*.AP (2016)		1.40	1.13–1.74	<0.01	0.575 6	0.758 7	100%
Omitting Reis *et al*.b (2017)		1.51	1.21–1.89	<0.01	0.616 0	0.784 8	100%
Omitting Reis *et al*.c (2017)		1.51	1.20–1.89	<0.01	0.618 0	0.786 1	100%
Omitting Salam *et al*.a (2005)		1.49	1.19–1.88	<0.01	0.642 0	0.801 3	100%
Omitting Salam *et al*.b (2005)		1.49	1.18–1.87	<0.01	0.650 9	0.806 8	100%
Omitting Salam *et al*.c (2005)		1.49	1.18–1.87	<0.01	0.651 9	0.807 4	100%
Omitting Salam *et al*.AP (2005)		1.48	1.18–1.87	<0.01	0.655 4	0.809 5	100%
Omitting Salihu *et al.* (2012)		1.50	1.19–1.88	<0.01	0.635 8	0.797 4	100%
Omitting Xu *et al*.a (2011)		1.46	1.16–1.84	<0.01	0.665 5	0.815 8	100%
Omitting Xu *et al*.b (2011)		1.46	1.16–1.84	<0.01	0.665 6	0.815 9	100%
Omitting Xu *et al*.c (2011)		1.46	1.16–1.85	<0.01	0.665 7	0.815 9	100%
Omitting Xu *et al*.AP (2011)		1.45	1.15–1.82	<0.01	0.659 4	0.812 0	100%
Omitting Zhou *et al*.a (2023)		1.47	1.17–1.86	<0.01	0.664 0	0.814 8	100%
Omitting Zhou *et al*.b (2023)		1.48	1.17–1.86	<0.01	0.662 0	0.813 6	100%
Omitting Zhou *et al*.c (2023)		1.47	1.17–1.86	<0.01	0.662 9	0.814 2	100%
Omitting Zhou *et al*.AP (2023)		1.47	1.17–1.86	<0.01	0.662 9	0.814 2	100%
Random effects model		1.46	1.16–1.84	<0.01	0.651 5	0.807 1	100%

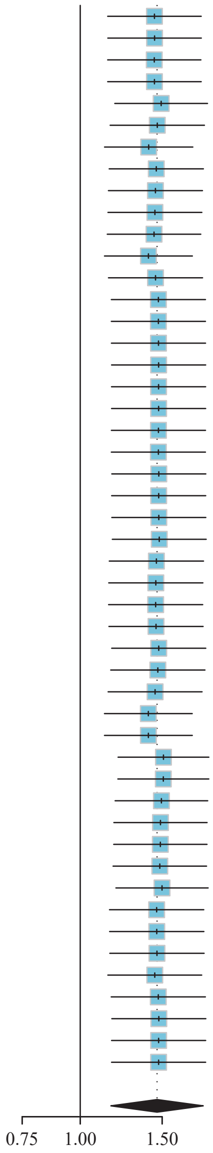

Supplementary Fig. 17 **Sensitivity analysis of pooled associations between PM_{10} exposure during the entire pregnancy and low birth weight risk (odds ratio [OR] with 95% confidence interval [CI]), analyzed using the random effects model.** a: first trimester, b: second trimester, c: third trimester, AP: entire pregnancy.

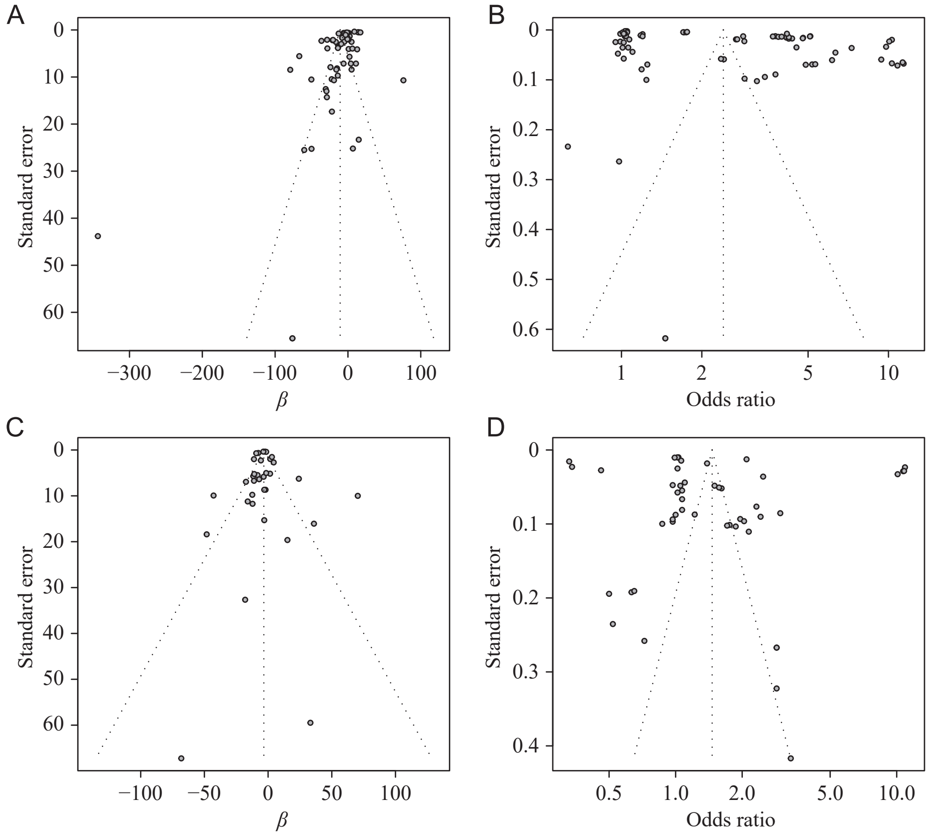

Supplementary Fig. 18 **Funnel plots of the meta-analyses assessing the associations between PM exposure and birth outcomes.** A: Changes in birth weight and $PM_{2.5}$. B: Changes in birth weight and PM_{10}. C: LBW risk and $PM_{2.5}$. D: Low birth weight risk and PM_{10}.
